# Determinants of fruit and vegetable consumption among children and adolescents: a review of the literature. Part I: quantitative studies

**DOI:** 10.1186/1479-5868-3-22

**Published:** 2006-08-11

**Authors:** Mette Rasmussen, Rikke Krølner, Knut-Inge Klepp, Leslie Lytle, Johannes Brug, Elling Bere, Pernille Due

**Affiliations:** 1 Department of Social Medicine, Institute of Public Health, University of Copenhagen, Denmark; 2 Department of Nutrition, Faculty of Medicine, University of Oslo, Norway; 3 Division of Epidemiology, University of Minnesota School of Public Health, Minneapolis, Minnesota, USA; 4 Department of Public Health, Erasmus University Medical Center, Rotterdam, The Netherlands

## Abstract

**Background:**

In order to more effectively promote fruit and vegetable intake among children and adolescents, insight into determinants of intake is necessary. We conducted a review of the literature for potential determinants of fruit and vegetable intake in children and adolescents.

**Methods:**

Papers were identified from Medline and PsycINFO by using all combinations of the search terms: "fruit(s) or vegetable(s)" and "children or adolescents". Quantitative research examining determinants of fruit and/or vegetable intake among children and adolescents aged 6–18 years were included. The selection and review process was conducted according to a four-step protocol resulting in information on country, population, design, methodology, theoretical basis, instrument used for measuring intake, statistical analysis, included independent variables, and effect sizes.

**Results:**

Ninety-eight papers were included. A large number of potential determinants have been studied among children and adolescents. However, for many presumed determinants convincing evidence is lacking, mostly because of paucity of studies. The determinants best supported by evidence are: age, gender, socio-economic position, preferences, parental intake, and home availability/accessibility. Girls and younger children tend to have a higher or more frequent intake than boys and older children. Socio-economic position, preferences, parental intake, and home availability/accessibility are all consistently positively associated with intake.

**Conclusion:**

The determinants most consistently supported by evidence are gender, age, socio-economic position, preferences, parental intake and home availability/accessibility. There is a need for internationally comparative, longitudinal, theory-based and multi-level studies taking both personal and environmental factors into account.

This paper is published as part of the special Pro Children series in the International Journal of Behavioral Nutrition and Physical Activity. Please see [] for the relevant editorial.

## Background

A large body of epidemiological evidence suggests that a high fruit and vegetable intake helps to promote health and to prevent chronic disease [1-4]. In most Western countries, large population groups, including children and adolescents, eat far less than the recommended amount of fruits and vegetables [5-8]. Several studies have shown that children's intake of fruit and vegetable tracks into adolescence [9-11] and that those food preferences and eating habits established in childhood and adolescence tend to be maintained into adulthood [12]. This makes increasing fruit and vegetable consumption among children and adolescents an important public health issue.

Interventions to improve health-related behaviours should be tailored to the most important determinants or mediators of these behaviours [13,14]. To date nutritional interventions have generally been only moderately successful in improving a lasting consumption of adequate amounts of fruits and vegetables [15]. To facilitate improvement of future interventions a comprehensive overview of the literature on determinants of fruit and vegetable consumption among children and adolescents is warranted.

**Figure 1:**
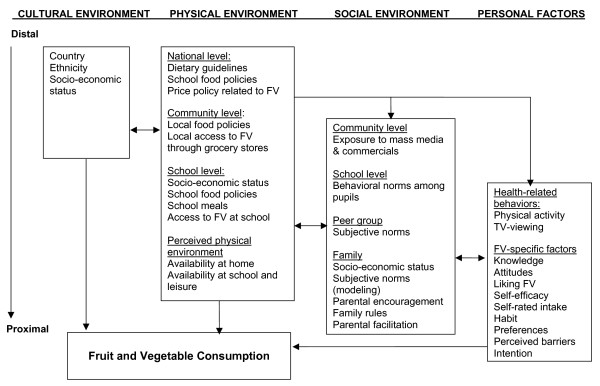
Conceptual framework applied to children's fruit and vegetable consumption: the Pro Children Project [16].

The present review is part of the Pro Children Project. The Pro Children Project is an international-study involving nine European countries aiming at assessing fruit and vegetable consumption among schoolchildren and their parents, as well as positively affecting determinants of children's consumption [16]. Based on constructs from different behavioural theories and the present review, a Pro Children conceptual framework considering both individual and environmental predictors has been developed (Fig. 1).

To our knowledge, a comprehensive review of the literature specifically studying determinants of children's fruit and vegetable intake has not previously been undertaken. The aim of the present paper is to provide a comprehensive review of potential determinants of fruit and vegetable intake in children and adolescents.

## Methods

### Literature search and data collection

Papers were identified by searching Medline (1966 to December 11, 2005) and PsycINFO (1806 to December 11, 2005). The search was conducted using all combinations of the search terms: fruit(s) or vegetable(s) and children or adolescents. The search from the databases identified 5,279 papers. The papers were screened by thorough reading of titles, abstracts, or full papers using the criteria for inclusion and exclusion specified below (table 1) by two co-authors (MR, RK and/or PD). Screening of titles and abstracts revealed 465 papers that were thoroughly read and considered for inclusion. Bibliographies of identified papers, former literature reviews and methodological papers, were searched recursively until no more papers emerged. MR, RK and PD were also primarily involved in the subsequent analyses of included papers. Any matter of doubt was discussed by at least two of the reviewers.

**Table 1:** Criteria for inclusion and exclusion

**Papers meeting inclusion criteria**
a) Investigate determinants of fruit and/or vegetable intake, either as the primary focus or as one of more outcomes
b) Identify fruit and vegetable consumption differentiated from other outcomes, either in a combined fruit and vegetable measure, as separate fruit and vegetable outcomes or as individual measures of specific fruits and vegetables
c) Are based on populations within an identifiable age-range of 6 to 18 years
d) Are population, community or school-based
e) Are based on research with humans

**Papers to be excluded are**

a) Qualitative papers
b) Papers reported in languages other than English
c) Review papers
d) Papers with methodological aims as the main purpose, for instance validation papers
e) Evaluation papers from interventions
f) Papers only describing prevalences, thus without analytical approach
g) Papers with analytical designs in which fruit and vegetable consumption is not clearly considered to be an outcome, but rather a determinant or a correlate without any hypothetical causal association (e.g. papers on BMI/fatness, dieting/weight control, other health behaviours – e.g. physical activity, smoking, and alcohol drinking)

### Analyses

The analytical approach followed a four-step protocol.

#### Step 1

For each paper meeting the inclusion criteria, a summary scheme containing study information on objective, study population, design, measurements of dependent and independent variables, statistical analyses, results, discussion, and potential bias was prepared. Information on the theoretical framework was also included. Specifically, it was recorded for each paper whether 1) the analyses were explicitly testing the relevance of one or more specified theoretical models, 2) the rationale for the analytical approach was based on a specified theoretical framework, or 3) the analyses were conducted without theoretical considerations. Some papers included multiple dependent variables or analysed multiple age groups. For these papers, the summary scheme contained information only for those variables and/or age-groups relevant for the present review.

#### Step 2

To evaluate the internal and external validity of the included papers, specific information on design and methodology were extracted and documented in a separate validity assessment scheme.

#### Step 3

An overall evidence scheme was produced including information on associations and significance for all variables involved in any paper. For each variable, the evidence was assessed across all papers included in the review. The identified variables were grouped into socio-demographic factors, personal factors, family-related factors, friends-related factors, school-related factors, meal patterns, TV watching, and eating fast food.

#### Step 4

To create a full overview of the included literature a summary table was constructed containing information on country, population, design, theoretical basis, instrument applied for measuring intake, statistical analysis, independent variables, significant associations identified, and effect sizes.

## Results

Ninety-eight papers matched all inclusion criteria and were included in the review. The first identified paper was from 1958. The review revealed a marked rise during the recent years in number of papers on determinants of children and adolescents' fruit and vegetable consumption.

From the evaluation of the design and methodology of each paper (table 2) the main findings are as follows:

**Table 2:** Validity assessment scheme: design and methodological characteristics of all included papers

**Characteristics**	**Reference no.**			
**World part/country**				

Asia	Bahrain: 17	Bangladesh: 18	China: 19, 20	India: 21
Europe	Belgium: 22, 23, 24, 25 Denmark: 26 England: 27, 28, 29, 30, 31, 32 Finland: 33, 34, 35	Greece: 36 Luxembourg: 37 Netherlands: 38 Northern Ireland: 39, 40	Norway: 41, 42, 43 Scotland: 44, 45, 46, 47 Sweden: 48, 49, 50, 51, 52	
North America	Canada: 53, 54 USA: 55, 56, 57, 58, 59, 60, 61, 62, 63, 64, 65, 66, 67, 68, 69, 70, 71, 72, 73, 74, 75, 76, 77, 78, 79, 80, 81, 82, 83, 84, 85, 86, 87, 88, 89, 90, 91, 92, 93, 94, 95, 96, 97, 98, 99, 100, 101, 102			
Oceania	Australia: 103, 104, 105, 106	New Zealand: 107, 108	Tasmania: 109, 110, 111, 112, 113	
South America	Costa Rica: 114			

**Design**				

Longitudinal	20, 39, 42, 46, 52, 59, 65, 79			
Cross-sectional	17, 18, 19, 21, 22, 23, 24, 25, 26, 27, 28, 29, 30, 31, 32, 33, 34, 35, 36, 37, 38, 40, 41, 43, 44, 45, 47, 48, 49, 50, 51, 53, 54, 55, 56, 57, 58, 60, 61, 62, 63, 64, 66, 67, 68, 69, 70, 71, 72, 73, 74, 75, 76, 77, 78, 80, 81, 82, 83, 84, 85, 86, 87, 88, 89, 90, 91, 92, 93, 94, 95, 96, 97, 98, 99, 100, 101, 102, 103, 104, 105, 106, 107, 108, 109, 110, 111, 112, 113, 114			

**Theoretical basis **(some papers may be marked more than once)				

Explicitly theory based	Social Cognitive Theory: 91, 93 Theory of Planned Behaviour: 79 The Stages-of-Change Trans-theoretical Model: 61			
Theoretical framework	Social Cognitive Theory: 41, 42, 43, 64, 67, 73, 75, 82, 86, 90, 102 Theory of Planned Behaviour: 22, 82 Social Learning Theory: 22 Problem Behaviour Theory: 43			
No theory applied	17, 18, 19, 20, 21, 23, 24, 25, 26, 27, 28, 29, 30, 31, 32, 33, 34, 35, 36, 37, 38, 39, 40, 44, 45, 46, 47, 48, 49, 50, 51, 52, 53, 54, 55, 56, 57, 58, 59, 60, 62, 63, 65, 66, 68, 69, 70, 71, 72, 74, 76, 77, 78, 80, 81, 83, 84, 85, 87, 88, 89, 92, 94, 95, 96, 97, 98, 99, 100, 101, 103, 104, 105, 106, 107, 108, 109, 110, 111, 112, 113, 114			

**Sample size **(individual level)				

Not reported	73			
< 500	18, 21, 22, 27, 33, 45, 50, 52, 53, 56, 62, 64, 66, 67, 71, 74, 77, 83, 88, 93, 95, 102, 107, 114			
500–1000	19, 20, 26, 31, 34, 39, 40, 42, 43, 46, 59, 63, 65, 72, 76, 92, 104, 105, 106, 108			
> 1000	17, 23, 24, 25, 28, 29, 30, 32, 35, 36, 37, 38, 41, 44, 47, 48, 49, 51, 54, 55, 57, 58, 60, 61, 68, 69, 70, 75, 78, 79, 80, 81, 82, 84, 85, 86, 87, 89, 90, 91, 94, 96, 97, 98, 99, 100, 101, 103, 109, 110, 111, 112, 113,			

**Age groups**				

Single age group	28, 30, 32, 35, 41, 43, 50, 56, 57, 61, 69, 71, 75, 76, 82, 86, 88, 93, 97, 105, 107, 108			
Multiple age groups/grades	17, 18, 19, 20, 21, 22, 23, 24, 25, 26, 27, 29, 31, 33, 34, 36, 37, 38, 39, 40, 42, 44, 45, 46, 47, 48, 49, 51, 52, 53, 54, 55, 58, 59, 60, 62, 63, 64, 65, 66, 67, 68, 70, 74, 77, 78, 79, 80, 81, 83, 84, 85, 87, 89, 90, 91, 92, 94, 95, 96, 98, 99, 100, 101, 102, 103, 104, 106, 109, 110, 111, 112, 113, 114			
Not specified	72, 73			

**Response rate **(individual level)				

Not reported	17, 18, 20, 21, 23, 24, 25, 31, 34, 36, 38, 40, 47, 49, 50, 53, 54, 60, 62, 64, 66, 71, 73, 74, 77, 78, 85, 88, 92, 93, 94, 95, 96, 100, 101, 103, 109, 113			
< 40%	29, 45			
40%–70%	22, 30, 52, 55, 56, 65, 70, 81, 84, 91, 99, 102, 104, 105, 107, 108			
71%–90%	28, 32, 35, 37, 39, 41, 42, 43, 44, 46, 48, 51, 58, 59, 63, 67, 68, 69, 72, 76, 80, 83, 86, 87, 89, 90, 97, 98, 110, 111, 112			
91%–100%	19, 26, 27, 33, 57, 61, 75, 79, 82, 106, 114,			

**Representative sample**				

Information not available in paper	20, 23, 27, 28, 34, 54, 55, 56, 59, 60, 65, 67, 69, 72, 73, 74, 75, 77, 83, 88, 91, 92, 93, 96, 97, 100, 106, 108			
Not representative beyond study population	19, 21, 22, 29, 31, 32, 40, 45, 47, 53, 57, 61, 62, 64, 68, 70, 71, 76, 78, 85, 86, 89, 90, 95, 103, 105, 107, 114			
Representative for restricted area	17, 18, 33, 46, 49, 51, 52, 63, 66, 79, 82, 84, 87,			
Representative for region/county	26, 30, 35, 37, 41, 42, 43, 48, 50, 99, 101, 102			
Representative for state/nation	24, 25, 36, 38, 39, 44, 58, 80, 81, 94, 98, 104, 109, 110, 111, 112, 113			

**Item descriptions**				

No detailed item descriptions (precise wording)	17, 18, 20, 21, 22, 24, 25, 27, 29, 30, 31, 34, 35, 37, 39, 40, 43, 45, 46, 47, 49, 50, 51, 52, 53, 54, 55, 56, 57, 58, 61, 62, 63, 64, 65, 66, 67, 68, 69, 71, 74, 75, 76, 77, 78, 80, 82, 83, 84, 86, 88, 89, 90, 91, 92, 93, 95, 99, 100, 102, 103, 105, 106, 107, 108, 109, 110, 111, 112, 113, 114			
Some items described in details (precise wording)	19, 23, 26, 28, 33, 38, 41, 59, 60, 70, 72, 73, 79, 81, 85, 87, 96, 97, 98, 101, 104,			
All items described in details (precise wording)	32, 36, 42, 44, 48, 94			

**Instrument for measuring F and/or V intake**				

FFQ	18, 19, 20, 21, 22, 23, 24, 25, 26, 28, 32, 33, 35, 36, 37, 38, 40, 41, 42, 43, 44, 46, 47, 48, 49, 50, 52, 53, 57, 59, 60, 61, 68, 69, 70, 72, 78, 79, 80, 81, 82, 84, 85, 86, 87, 94, 95, 96, 97, 98, 99, 100, 101, 102, 106, 108, 109, 113			
24-h-recall	17, 34, 58, 66, 71, 75, 76, 83, 88, 92, 93, 103, 104, 105, 107			
Food diary/record	27, 29, 30, 31, 45, 55, 64, 67, 73, 74, 77, 91, 110, 111, 112, 114			
Others	39, 51, 56, 62, 63, 65, 89, 90			
Information not provided	54			

**Differentiation of outcome measure**				

Single F and/or V items	47, 53, 108, 109, 113			
Only fruit	24, 25, 38, 40, 62, 88			
Only vegetables	35			
F and V separately	17, 18, 21, 22, 23, 26, 27, 29, 31, 33, 34, 36, 37, 39, 44, 45, 46, 48, 49, 50, 51, 52, 56, 60, 63, 65, 66, 68, 69, 76, 77, 78, 83, 85, 87, 89, 95, 97, 98, 100, 103, 104, 105, 106, 107, 112, 114			
F and V combined	19, 20, 28, 30, 32, 41, 42, 43, 57, 58, 61, 64, 70, 71, 75, 79, 80, 81, 82, 84, 86, 90, 91, 93, 94, 96, 101, 102, 110, 111			
Separately and combined	54, 55, 59, 67, 72, 73, 74, 92, 99			

**Validity of applied measurements**				

No information	17, 20, 24, 27, 31, 36, 39, 46, 47, 49, 57, 58, 60, 71, 74, 76, 77, 78, 81, 84, 98, 103, 105, 106, 107, 114			
Only reference(s) to former publications	21, 23, 25, 29, 30, 32, 44, 45, 50, 51, 52, 53, 54, 56, 59, 65, 68, 69, 70, 72, 80, 83, 87, 88, 89, 92, 99, 104, 110, 111, 112			
Validity assessed for all or some items/scales within the applied study population	18, 19, 22, 26, 28, 33, 34, 35, 37, 38, 40, 41, 42, 43, 48, 55, 61, 62, 63, 64, 66, 67, 73, 75, 79, 82, 85, 86, 90, 91, 93, 94, 95, 96, 97, 100, 101, 102, 108, 109, 113			

**Analysis**				

Uni-variate (including stratified analyses)	18, 21, 29, 30, 31, 33, 34, 36, 37, 39, 40, 45, 46, 47, 51, 53, 57, 60, 65, 78, 83, 89, 90, 94, 95, 101, 105, 106, 108, 109, 110, 111, 112, 114			
Multivariate	19, 20, 22, 23, 24, 25, 28, 32, 35, 38, 41, 42, 43, 44, 48, 49, 50, 52, 55, 56, 58, 59, 61, 63, 64, 66, 67, 68, 69, 70, 72, 73, 75, 76, 79, 80, 81, 82, 84, 85, 86, 87, 91, 92, 93, 96, 97, 98, 99, 100, 102, 103, 104, 113			
Descriptive	17, 26, 27, 54, 62, 71, 77, 88, 107			
Not specified	74			

F = fruit; V = vegetables; FFQ = food frequency questionnaire; 24-h recall = 24 hour recall

• Forty-eight (49%) of the included papers are based on US study populations

• Eight papers (7%) use data from longitudinal studies

• Four papers (4%) are explicitly testing the relevance of a theory while 12 papers (12%) are based on a theoretical framework

• In 24 papers (25%) the sample is smaller than 500 individuals

• Within 81 papers (83%) the samples are not representative for the age group at large in the country of study, or insufficient information is available for assessing representativeness.

• In 58 papers (59%) the instrument applied for measuring fruit and/or vegetable intake is a food frequency questionnaire; 15 papers (15%) use one or several 24-hour-recalls; 16 papers (16%) use food diaries/records. Nine papers (9%) use other instruments, e.g. 'eating fruits and vegetables on the previous day (yes/no)' and 'type of food eaten for snack'.

• Six papers (6%) analyse only fruit intake and one paper (1%) is on vegetable intake solely. In 47 papers (48%) fruit and vegetable intake is analysed separately. Thirty papers (31%) analyse a combined measure of fruit and vegetable intake while nine papers (9%) include analyses on both separate and combined measurements. Consumption of single items of specific fruit and/or vegetables (e.g. apples, tomatoes etc.) is analysed in five papers (5%).

• Twenty-six papers (27%) are characterised by providing no information on validity of applied measurements and 31 papers (32%) only provide reference(s) to former publications. In 41 papers (42%) validity is assessed for all or some items/scales applied.

• In 54 papers (55%) multivariate analyses were conducted while 34 (35%) papers include univariate analyses. Nine papers (9%) only include descriptive analyses. For one paper (1%) the type of analysis is not specified.

Table 3 summarises associations between potential determinants of fruit and vegetable consumption among children and adolescents that were examined in at least three papers. The determinants were grouped into socio-demographic factors, personal factors, family-related factors, friends-related factors, school-related factors, meal patterns, TV watching, and eating fast food.

**Table 3:** Summary of potential determinants of fruit and vegetable consumption among children and adolescents. Determinants included in at least three papers.

**Determinant variable**	**Association/group with highest level of intake (Reference no.)**	**No association (Reference no.)**
**Sociodemographic factors**		

Gender	Girls: 19, 23, 24, 25, 27, 30, 36, 37, 43, 44, 46, 47, 48, 50, 52, 53, 55, 63, 77, 85, 92, 100, 105, 106, 107, 108, 109 Boys: 17, 60, 110, 114	26, 28, 40, 57, 65, 67, 74, 77, 80, 82, 84, 90, 98, 99, 101, 103, 111, 112
Age/grade	Neg. assoc: 23, 24, 25, 36, 37, 40, 63, 80, 98, 113 Pos. assoc: 110, 111, 112	17, 66, 67, 77, 82, 85, 99, 101, 103
Socioeconomic position *(some papers included several measurements and they may therefore be marked twice)*	Pos. assoc: 18, 19, 21, 23, 24, 25, 27, 32, 33, 34, 35, 37, 39, 40, 43, 44, 46, 47, 48, 49, 50, 52, 54, 60, 63, 66, 73, 80, 82, 85, 86, 98, 100, 104, 105, 108, 112 Neg. assoc: 20, 60, 84	18, 19, 20, 24, 28, 33, 34, 50, 55, 63, 66, 74, 80, 82, 99, 100, 103, 110, 111
Race/ethnicity	Assoc: 26, 28, 57, 60, 65, 77, 78, 80, 84, 85, 90, 92, 98, 100, 101	55, 63, 67, 74, 82, 99
Urbanisation	Rural: 20, 47, 114	19

**Personal factors**		

Preferences	Pos. assoc: 41, 42, 56, 63, 67, 73, 82, 86, 91, 109, 113	-
Nutritional knowledge	Pos. assoc: 26, 41, 42, 48, 75, 93 Neg. assoc: 82	91
Attitudes	Pos. assoc: 22, 79, 86	-
Intentions	Pos. assoc: 22, 79	41, 42
Self-efficacy	Pos. assoc: 22, 41, 42, 67, 75, 102	86, 91
Outcome expectations	Pos. assoc: 91	67, 73, 75, 82
Perceived barriers	Low barriers: 79	66, 82
Subjective norms (perception of others' attitude on own diet)	Pos. assoc: 43, 79, 82	

**Family-related factors**		

Parental intake	Pos. assoc: 22, 41, 45, 72, 75, 102, 109, 113	42
Home availability/accessibility	Pos. assoc: 41, 42, 64, 72, 73, 75, 86, 93, 102	41, 64
Family structure	Two-parent family: 35, 84, 99, 101	82
Family size		19, 110, 111
Frequency of family meals	Pos. assoc: 35, 70, 86, 87, 98	22
Parental style	Assoc: 38, 82, 101	22, 43, 102
Parental support for healthy eating/FV	Pos. assoc: 22, 86, 102	-

**Friends-related factors**		

Perceived friend intake	Pos. assoc: 22, 113	109

**School-related factors**		

Fruit and/or vegetable availability at school	Pos. assoc: 42, 73	24
Other foods: Vending machines	Neg. assoc: 76	31, 24
Other foods: À la carte program availability	Neg. assoc: 76	-
Other foods: Access to snack bar meals	Neg. assoc: 63, 65	-
Participating in school lunch programme	Pos. assoc: 35, 77, 84	-
Academic achievement	Pos. assoc: 35, 85	43
School type	Non-public schools: 89, 112	110, 111

**Meal patterns/TV watching/eating fast food**		

Meal frequency	Pos. assoc: 43	66, 86
Meal type	Pos. assoc: 55, 62, 71, 83, 88	-
Hours of TV watching	Neg. assoc: 36, 59, 81, 97	-
Eating fast food	Neg. assoc: 58, 68, 96	86

Pos. assoc = positive association; neg. assoc. = negative association; F = fruit; V = vegetables

### Socio-demographic factors

#### Gender

Gender differences in fruit and vegetable consumption were studied in 49 papers. Twenty-seven of these found that girls tend to have a higher or more frequent intake of fruit and/or vegetables than boys. Eighteen papers observed no differences between boys and girls and four papers observed the highest or most frequent intake among boys. No systematic differences in age groups or instrument used for measuring fruit and/or vegetable intake exist between papers that identified gender differences in fruit and vegetable consumption and those that did not. However, a systematic difference seems to exist according to geographical region. The majority of the papers that analysed the effect of gender were either from the US or Europe. Of the 18 US papers only six found a difference in intake among boys and girls while 14 of the 17 European papers identified a gender difference. Thus, gender differences in intake of fruit and vegetables seem to be a phenomenon more prevalent among children and adolescents in European countries compared to children and adolescents in the US.

#### Age/grade

The relevance of age (or grade) was studied in 22 papers. Ten of these papers found that fruit and vegetable consumption decreases with increasing age. In nine papers no effect of age was observed. In general, these nine papers are not characterised by narrower age/grade ranges or more extensive confounder control than those papers that did identify an association between age and fruit and vegetable intake. However, the majority of the papers that identified a negative association for age measured fruit and vegetable intake by a food frequency questionnaire (nine out of ten papers) while 24-hour recalls/food records were the dominant instrument applied among the papers that did not identify an association for age (six out of nine papers). This finding suggests that all age groups may eat the same amounts of fruit and vegetables but that the younger age groups eat fruit and vegetables more frequently than the older age groups or that there is an age-related response bias by assessment instrument used. Additionally, all of the six European papers that analysed the effect of age observed a negative association. In contrast, most of the US papers that analysed the effect of age found no association with intake (seven out of ten papers). Thus, decreasing intake of fruit and vegetables with increasing age of children and adolescents seems to be more related to European societies than to the US. Three papers found a positive association between age and intake of fruit and vegetables. In these three papers slightly different analyses were conducted on the same Tasmanian data set.

#### Socioeconomic position

Forty-six of the identified papers examined the influence of socioeconomic position (SEP). Within these papers, a large number of operationalisations of SEP were applied. Still, the findings are consistent. Low SEP is associated with low or less frequent intake of fruit and vegetables, and especially for family income (positive association in seven out of 14 papers), parental occupation (positive association in nine out of 11 papers) and parental education (positive association in 11 out of 11 papers) the association is well documented. For father's occupation specifically, two papers found a positive association, two papers found no association, and one paper found that high SEP students ate fruit more frequently at home while the most frequent intake of fruit at school was seen among low SEP students. The influence of mother's occupation was studied by two papers. Both found no association with fruit and vegetable consumption. The specific influence of father's education was studied in six papers of which only one identified a positive association. The influence of mother's education was analysed in eight papers of which four found higher or more frequent intake of fruit and/or vegetables with increasing educational level of mother. One longitudinal study, conducted in China, reported a negative association for mothers' educational level. Other examples of operationalisations of SEP that have been included in papers on fruit and vegetable consumption among children and adolescents are family material affluence (positive association in three out of four papers), student educational status (positive association in three out of three papers), several combined measurements (negative association in one out of two papers), and contextual measurements based on neighbourhoods or school catchments areas (positive association in six out of ten papers). No general systematic differences in age of study populations exist between those papers that identify an association between SEP and intake of fruit and/or vegetables among children and adolescents and those papers that do not. Also no clear systematic differences seem to exist according to geographical region. This is partly due to the fact that a number of papers each include two or more separate measurements for SEP for which different associations with intake of fruit and vegetables are observed.

#### Race/ethnicity

The literature on the influence of race/ethnicity is highly dominated by studies conducted with a US study population. The significant associations found (13 out of 19 studies) are inconsistent, depending on which ethnic groups were compared and often different patterns of consumption were observed for fruit and vegetables.

In addition, 11 of the papers on race/ethnicity differences do not adjust for SEP. Comparing results may therefore be difficult due to differences in adjustments for confounders. Two European papers on the influence of race/ethnicity have been identified. One British paper (adjusted for SEP) observed the most frequent intake of fruit and vegetables among Black adolescents compared to White and Asian adolescents. One Danish paper (unadjusted analyses) found that immigrant children (not specified) consume fruit and vegetables more frequent than Danish children.

#### Urbanisation

The influence of urbanisation has been investigated in four papers. Three of these found that fruit and/or vegetable consumption is higher or more frequent among rural children and adolescents than among urban children and adolescents. One paper found no association.

### Personal factors

#### Preferences

Preference is the personal factor that has been examined most extensively. Eleven papers analysed the influence of preferences, and in all 11 papers a positive association between preferences and children and adolescents' intake of fruit and/or vegetables was observed.

#### Other personal factors

For nutritional knowledge, six papers observed positive associations with fruit and/or vegetable intake, one paper observed a negative association, and one paper did not find any association. Positive associations were also observed for attitude (three out of three papers), intentions (two out of four papers), self-efficacy (six out of eight papers) and subjective norms (perception of others' attitude on own diet) (three out of three papers). Five papers analysed the influence of outcome expectations and in one paper a positive association was identified. Barriers for eating fruit and vegetables were studied in three papers by the use of combined measures, e.g. availability, self-efficacy, cost, taste, and appeal. One paper found the most frequent intake of fruit and vegetables among adolescents with low perceived barriers. Two papers found no association.

### Family-related factors

#### Parental intake

Within the family arena, a positive association between parental intake of fruit and/or vegetables and children's fruit and/or vegetable consumption was observed in eight out of nine papers. Four of these papers analysed parent reported parental intake and six papers analysed child perceived parental intake (one paper analysed both child-and parent-reported parental intake). One of these papers included perceived parental intake in a combined measure of perceived intake of significant others (friends, home-economics teacher, and/or siblings). Within two of the papers the effect of parental intake was modified by home availability, i.e. there is a stronger positive association between parental intake and children's fruit and vegetable intake within families with a high availability of fruit and vegetables at home.

#### Home availability and accessibility

Three out of three papers identified a positive association between child-reported home availability of fruit and vegetables (fruit and vegetables being present in the home) and children's intake of fruit and vegetables. Within one of these papers, the association was only observed among girls. The influence of parent-reported availability was also analysed by three papers. One paper found no association, one paper found a positive association for girls only, and one paper found a direct positive association for girls and an indirect effect for boys (through motivation operationalised by a combined measure of self efficacy, outcome expectancies and preferences). One out of one paper observed a positive association for parent-reported home accessibility of fruit and vegetables (form and location of fruit and vegetables present in the home). Within the same paper, a positive association was identified for child-reported accessibility among girls. Two out of three papers observed a positive association for combined measures of parent-reported home availability and accessibility. Combined measures of child-reported home availability and accessibility was analysed in only one paper. Here, a positive association was identified.

#### Family structure

The influence of family structure on children's fruit and vegetable consumption was analysed in five papers. Four papers found that fruit and/or vegetable consumption is lower among children from single-parent families than among children from two-parent families. Within two of these papers the effect of family structure were not adjusted for SEP. One paper (adjusted for SEP) found no association.

#### Family size

The influence of family size was studied in three papers which all found no association with children's fruit and vegetable consumption.

#### Family meals

Six papers studied the influence of shared family meals and within five of these a positive association with children's consumption of fruit and/or vegetables was observed. One paper found no association.

#### Parenting style

The association between parenting style and children's intake of fruit and vegetables was studied in six papers. These papers included ten different operationalisations of parenting styles. Three papers identified an association with children's fruit and vegetable consumption. In these papers the highest or most frequent intake was seen for authoritative parenting style, maternal authoritative parenting style, paternal non-authoritative style, and parental monitoring.

#### Parental support for eating fruit and vegetables/healthy eating

Three out of three papers found a positive association between fruit and/or vegetable consumption and parental support for eating fruit or vegetables/healthy eating.

### Friends-related factors

The influence of friends has been only sparsely investigated. Two out of three papers found that perceived friend intake is positively associated with fruit and/or vegetable consumption.

### School-related factors

#### Availability and policy of healthy and unhealthy foods

A variety of different operationalisations of availability of fruit and vegetables and other foods at school have been analysed in seven papers. One US and one Norwegian paper found a positive association for fruit and vegetables being served as part of the school lunch/fruit being served as part of a school fruit programme. No association was found between school availability of fruit (sold in school stores) and students' fruit consumption in a study from Belgium-Flanders. The same study also found no association for vending machines (selection not specified) being present at school. Another paper found that the number of snack vending machines (not differentiated according to snack type) is negatively associated with students' intake of fruit and vegetables. A limited number of papers also showed that à la carte school lunch program availability is negatively associated with students' intake of fruit and vegetables (one out of one papers), and that fruit and vegetable intake is lower among students with access to snack-bar meals versus those students with access to school lunch meals (two out of two papers).

Two US and one Finnish paper found that students who participate in school lunch programmes have a higher or more frequent intake of fruit and vegetables than students that do not participate.

#### Student-school relations

The influence of academic achievement was studied by three papers. In two of these, low academic achievement is associated with low frequency of fruit and vegetable consumption.

#### School type

The influence of school type was studied in four papers (unadjusted analyses) of which two found that students from non-public schools have the highest or most frequent intake of fruit and/or vegetables.

### Meal patterns

The literature on the influence of meal patterns includes different measurements. In one out of three papers meal frequency is positively associated with fruit and vegetable consumption (meals are not differentiated according to type or content). Significant associations for meal type were found in five out of five papers though the applied operationalisations varied (type of meal during day (e.g. breakfast and lunch), school breakfast vs. home breakfast, and weekday meals vs. weekend day meals).

### TV watching

Four papers examined the effect of watching TV, and they all found that increasing hours of TV watching is associated with decreasing fruit and/or vegetable consumption.

### Eating fast food

The influence of eating fast food was studied in four US papers. Of these, three papers found that eating fast food is associated with low or less frequent consumption of fruit and vegetables, while one paper found no association

Table 4 summarises associations between potential determinants of fruit and vegetable consumption among children and adolescents that were examined in less than three papers. From this table it is evident that especially the literature on family-related factors has investigated a variety of potential determinants that each has only been included in one or two papers.

**Table 4:** Summary of infrequently tested determinants of fruit and vegetables consumption among children and adolescents. Determinants included in less than three papers.

**Determinant variable**	**Association/group with highest level of intake (Reference no.)**	**No assoc (Ref. no.)**
**Sociodemographic factors**		

Country (European)	Southern European countries: 25	
Student working status	Non-working: 95	
Type of dwelling		50

**Personal factors**		

Evaluation of own diet	Pos. assoc: 79	
Motivation (self-efficacy, outcome expectations, preferences)	Pos. assoc: 93	
Sweetened beverage preferences		66
Bottled water preferences		66
Stages of change	Action/maintenance stage: 61	
Asking/behavioural skills		41, 91
Stress	Low levels of stress: 28	
Subjective health complaints		43
Depression	Low level of depression: 28	69
Outlook for the future		82
Negative self-evaluation		79
Evaluation of own health		79
Perceived healthiness		109, 113
Spirituality	Pos. assoc: 82	
Exposure (former eating of fruit and vegetables)		91

**Family-related factors**		

Number of children in the family		50
Place of residence	Living outside family: 46	
Social norms (perception of others attitude on eating FV)		91
Positive relations with parents	Pos. assoc: 22, 43	
Family connectedness	Pos. assoc: 85	
Family cohesion		22
Family adaptation		22
Family communication	Pos. assoc: 101	
Frequency of communication of dislike		22
Use of positive strategies for communication of dislike		22
Use of negative strategies for communication of dislike	Neg. assoc: 22	
Number of hours spent without parents	Neg. assoc: 101	
Parents present when child leaves and returns from school		98
Shared shopping		22
Children ask for healthy food to be brought from store	Pos. assoc: 22	
Food asked for is bought	Neg. assoc: 22	
Children preparing their own meals	Neg. assoc: 84	
Food decision-making (parent/adolescent)		98
Parents prepare high/low-fat foods		66
Home high/low-fat availability		66
Mean home high/low-fat practice		66
Parental knowledge	Pos. assoc: 75	
Parental self-efficacy to serve FV	Pos. assoc: 75	
Parental smoking	Neg. assoc: 29	43
Parental physical activity		43
Expenditures on food	Neg. assoc: 18	

**Friends-related factors**		

Positive relations with friends	Pos. assoc: 43	

**School-related factors**		

Using the canteen	Non-canteen users: 103	
Lunch source	Bringing lunch from home: 99	
School food rules		24
Nutritional education		24
School size		55
Percent of Euro-American students at school	Pos. assoc: 55	
Percent of students at school participating in school lunch	Pos. assoc: 55	
Annual out-migration rate		55
Liking school	Pos. assoc: 35	43
Antisocial behaviour		43

**Meal patterns/TV watching/eating fast food**		

Having regular meals	Pos. assoc: 51, 94	
Skipping meals	Neg. assoc: 84	
Number of snack meals		66
Snacking during school day	Neg. assoc: 35	
Watching TV while eating	Neg. assoc: 83	
Computer use	Pos. assoc: 97	
Reading/doing homework	Pos. assoc: 97	

Pos. assoc. = positive association; neg. assoc. = negative association; F = fruit; V = vegetables

A comprehensive summary table (table 5) containing detailed information on design, analysis, and results of included papers is electronically available [115].

## Discussion

The research presented in this review shows that the scientific effort to identify determinants of fruit and vegetable consumption among children and adolescents has increased substantially over the past four decades growing from a total of 11 published papers from 1958 through 1986 to 54 papers published only since 2000. This increase follows the growing evidence supporting the health benefits from eating ample amounts of fruit and vegetables [1-4].

This review of determinants of fruit and vegetable consumption among children and adolescents reveals that the determinants supported by the greatest amount of evidence are gender, age, SEP, preferences, parental intake, and home availability/accessibility. Girls tend to have a higher or more frequent intake of fruit and vegetables than boys, and a corresponding pattern is seen for the younger age groups compared to the older age groups. As mentioned, solely descriptive prevalence papers were not included in this review. Such papers may contain information on age and gender differences. Therefore more documentation in this area might have been gained by including prevalence papers. SEP, preferences, parental intake, and home availability/accessibility are all positively associated with children and adolescents' fruit and vegetable consumption. In addition, also for nutritional knowledge, self-efficacy and shared family meals the evidence for positive associations is rather convincing.

A large number of potential determinants have been included in the quantitative scientific literature on fruit and vegetable consumption of children and adolescents. However, this review clearly shows that for many variables evidence is lacking. For the majority of the examined variables this lack of evidence is mainly due to lack of studies. For a minority of the identified variables (e.g. gender, age and SEP) a significant number of studies exist. Here, the literature is generally characterised by conclusive and similar findings within a large part of the papers and non-significant associations identified in the remaining papers. Only rarely conflicting results were seen.

Establishing epidemiological evidence for a given association implies the existence of only few cases of contradictory findings. Though being low in numbers, it is still important to analyse reasons for contradictory findings. Naturally, such findings may be due to methodological bias while others may reflect true differences between countries, geographical regions, time periods etc. Thus, contradictory findings should be seriously considered, as they may provide important information needed for creating new scientific hypotheses.

For variables studied in at least three papers included in the present review contradictory findings were observed for gender, age, SEP and nutritional knowledge.

In contrast to most of the papers analysing the influence of gender, four studies observed the highest or most frequent intake of fruit and/or vegetables among boys. Of these four papers, one US study by Burdine et al. (1984) [60] and one Tasmanian study by Woodward (1985a) [110] were both rather old publications compared to the majority of the included papers. The two remaining papers by Musaiger and Gregory (1992) [17] and Rojas (2001) [114] analysed study populations from Bahrain and Costa Rica (small sample). No other papers from South America and only five Asian papers were identified. The contradictory findings identified for gender may be due to methodological bias or they may reflect true associations existing in US and Tasmanian societies in the mid nineteen eighties. Finally, the two last contradictory findings may reflect true situations in geographical areas less investigated.

Three papers observed a positive association between age and intake of fruit and vegetables. As earlier described these three papers are based on the same Tasmanian data set. Data was collected in the early nineteen eighties, and it is therefore uncertain if these differences would still be observed at present.

In contrast to most of the identified papers analysing the association between SEP and fruit and vegetable consumption among children and adolescents, three papers observed the highest or most frequent intake of fruit and vegetables among low SEP groups. One US study by Melnik et al. (1998) [84] of 2^nd ^and 5^th ^grade students in the city of New York found that 5^th ^grade students from low SEP households more frequently consumed fruit and vegetables than students from high/medium SEP households. Categorisation of SEP groups was based on a measure combining information on number of parents working, eligibility for free or reduced price school lunch, and use of federal assistance programs. The contradictory finding is not discussed within the paper. However, the study is characterised by an individual level response rate of 51%, which may have introduced a selection bias by which the low SEP participants are not representative for low SEP households in the city of New York. Another US study by Burdine et al. (1984) [60] of 7^th ^and 8^th ^grade students from Texas investigated determinants for fruit and vegetable consumption at home and at school respectively. Based on father's occupation, this study found that high SEP students ate fruit more frequently at home, while the most frequent intake of fruit at school was seen among low SEP students. As argued in the paper, these findings suggest that school nutrition subsidies may provide more opportunities for low SEP students to eat healthful foods. The division of total intake measures into measurements of what is consumed at home versus in school may be a new and important aspect to consider for future studies. Using a more general measure for intake that is not calculated by time of the day or different settings may hide important information. Finally, a longitudinal Chinese study by Wang et al. (2002) [20] found that children whose mothers had higher educational levels were less likely to maintain a high fruit and vegetable diet. Within the paper it is argued that although these mothers were likely to have better access to the media and to health- and nutrition-related knowledge, their behaviour indicates that they are not aware of the health concerns related to higher fat foods. At the same time these families may also have more family resources, by which they can afford more expensive foods, such as meats and cooking oil. The authors also argue, that another possibility is that these women understand the need for energy-dense diets linked with high meat and fat intake and promoted concern for growth and development over the concerns for obesity and diet-related non-communicable disease. Conclusively it is stated that the findings from China suggest that mothers' nutritional knowledge, health consciousness and exposure to the media may influence their children's diet beyond the determining role of family resources and access to foods available to the community in developing countries undergoing a rapid social and economic transition. The present review only identified very few studies conducted in developing countries, and the results from the Chinese study exemplify the importance of initiating future studies involving study populations from countries with varying levels of developmental stages and with different political systems.

For nutritional knowledge, one US study by Lytle et al. (2003) [82] found that students in the 25^th ^percentile for the knowledge score reported significantly greater intake of fruit and vegetables compared to students in all other quantiles. As stated in the paper, this finding may be due to a ceiling effect as most students scored very high on the knowledge questions.

In conclusion, several of the few contradictory findings identified within the present review may be attributable to methodological bias, while others may reflect realities.

No previous review comparable to the present review exists. Recently a systematic review was conducted by Blanchette & Brug (2005) [116]. This review is restricted to the years 1990 to March 2005 and it focuses on determinants of fruit and vegetables consumption among 6–12 year-old children with emphasis on factors easily influenced through intervention. Therefore socio-demographic factors as ethnicity, gender, and SEP are not included. Past reviews have generally been characterised by either having a broader focus in terms of food categories (e.g. general eating behaviours and food patterns) [e.g. [13,117]] or by being focused on a specific group of determinants (e.g. family and television watching) [118-120]. The majority of the previous reviews did not aim at presenting a total overview of the literature on determinants. Rather, the aim has been to conduct an exploratory and non-exhaustive search to develop, understand and/or present a conceptual framework for understanding adolescent eating patterns and/or to inspire future development of interventions, policies and research [e.g. [13,117,121,122]]. The aim of the present review was to conduct a comprehensive and exhaustive search from which the evidence of specific potential determinants of a specified food category, namely fruit and vegetables was systematically evaluated based on standardised procedures. Due to these marked differences in purpose and methodology it is therefore difficult to make detailed comparisons between the present review and related previous reviews.

This review has several strengths and limitations. Literature relevant for the present review was identified by searching Medline and PsycINFO. Inclusion of other databases may have led to identification of additional relevant papers that matched the inclusion criteria. For instance, it is rather surprising that no papers on industry promotions (e.g. advertising and marketing of fruit and vegetables or competitive food choices as chips, chocolate bars etc.) as a determinant of fruit and vegetable intake in children and adolescents were identified by the search and selection criteria. However, bibliographies of relevant papers including reviews and methodological papers were searched thoroughly until no more relevant papers emerged. Due to the language criteria, relevant information published in other languages than English may have been missed. The review is strengthened by the systematic approach by which procedures for evaluation and categorisation of all included papers were standardised. Within this review only significant associations are considered. In the field of epidemiology, the criteria for evaluation of estimated associations is a topic of discussion, and one recommendation is not to base evaluations on statistical significance alone [123]. Preferably, both the significance and the magnitude of association of all included potential determinants should be evaluated. However, this has not been feasible as several papers only report estimates for significant associations or simply levels of significance without estimates of the associations.

Adolescence is a time period of rapid physical maturation and growth combined with a psychological and social development which is often accompanied by changes in social influences. As children move into adolescence family influences often decrease due to competing influences from other social settings [124]. It could therefore be hypothesised that differences in determinants of fruit and vegetable consumption exist between children and adolescents. The present review includes children and adolescents in the age range of 6 to 18 years. It is therefore rather surprising that for the variables investigated most extensively, no differences in age exist between papers observing significant associations and papers that do not. The rest of the variables (the majority) included in this review are characterised by both being sparsely investigated and by showing few contradictory findings. Differentiating the results for these variables in relation to age is therefore hardly possible.

### Methodological and design issues

There has been a clear quality improvement of the literature on determinants of fruit and vegetable consumption among children and adolescents over the past 15 years. In recent years a number of papers with high internal and external validity have been published [e.g. [25,35,41,58,79,87]]. However, our analyses of all papers included in this review revealed some general issues on design and methodology that affect the validity of the generated results as well as the possibilities for comparing results from different papers.

A considerable part of the papers include analyses based on small study samples and many papers include samples that are non-representative or only representative of a restricted geographical area. Often the validity of the applied instruments are only considered very superficially or not mentioned at all. Insufficient confounder control also seems to be a problem in a number of papers. For example, in some papers the analyses are not adjusted for relevant socio-demographic factors such as gender, age and/or SEP. Other bias may be evident in the types of instruments used (recalls versus food frequencies) by age of participant. Parts of the literature on determinants of fruit and vegetable consumption among children and adolescents are therefore characterised by several methodological problems that may affect internal and external validity of the generated results.

Comparisons of results from the different papers may be problematic for several reasons. A large variety of approaches for conceptualising, operationalising, measuring, and coding the outcome variable(s) exists among the identified papers. Some papers consider frequency of intake, while others consider amount of intake, and while these may be inter-correlated and both lead to the desired enhancement of intake, they may have different determinants. For instance, increasing diversity of fruit in the child's home may increase the amount of intake, while it may have no influence on frequency of intake, if measured as daily versus less than daily. In contrast, it may also be hypothesised that an increase in frequency of intake may be accompanied by decreasing portions sizes. In terms of defining the outcome variables, the possibility for comparing results is also compromised by two other aspects: 1) In some papers fruit consumption and vegetable consumption is analysed as separate outcomes, whereas others conduct the analyses on one combined measure. Comparing results from papers applying different analytical approaches may therefore be difficult. Additionally, combined analyses of fruit and vegetable intake may hide the fact that eating fruit and vegetables respectively may be linked to different sets of determinants; 2) Comparing results from different papers may also be problematic due to the fact that some papers include potatoes in the vegetable measurement, while others do not. In almost half of the included papers that analyse vegetable consumption, insufficient information is provided to assess whether potatoes are included in the measurement of vegetable intake or not. It is therefore not possible to evaluate the bias introduced by different definitions of vegetables, when comparing results. Correspondingly, some papers include fruit juice in the fruit category, while others do not. In more than half of the included papers it is not possible to assess, whether fruit juice is included in the measurement of fruit consumption.

### Conclusion and recommendations for research

The conceptual framework applied within the Pro Children project is one of the most comprehensive models applied within the research on fruit and vegetable consumption among children and adolescents. The present review shows that several areas in this model can be identified for which research is very limited or lacking:

• Psychosocial behavioural theories have been applied most often. Still, relatively few personal factors have been analysed extensively.

• Family-related factors have been investigated most extensively. However, this part of the literature is characterised by a large number of conceptually different factors that are each investigated only sparsely. Generally, and also in terms of establishing healthy food habits, parents function as important role models for their children. Parents are responsible for making healthy food items available and accessible to the child within the home and for supporting and encouraging the child to make healthy food choices overall. However, the present review shows that only for a very limited number of family-related factors, good evidence exists. To enable health promoters to make evidence based decisions, more studies on the influence of the family setting for influencing fruit and vegetable intake among children and adolescents are therefore needed.

• An overview of the effectiveness of interventions conducted at schools to promote fruit and vegetable intake among school children reveals that multi-component approaches including active provision of fruit and vegetables at lunch are those approaches that have been most successful [15,125]. Still, considering the fact that most interventions aiming at promoting fruit and vegetable intake among children and adolescents are conducted at school the number of observational (non-experimental) studies of potential school-related determinants are surprisingly low. Non-experimental studies on the association between school food environments and policies and adolescent eating patterns have been conducted [e.g. [126]] but studies specifically analysing fruit and vegetable consumption are still lacking.

• Papers on potential influences of national level factors are almost absent. There is an obvious lack of papers looking at international differences in predictors. Future international comparative surveys should enable investigations of national level factors of importance e.g. price levels, policy, guidelines, supply, and exposure to mass media and commercials.

• Likewise, little research has been reported on the potential influence of community or neighborhood level factors. Future research should therefore study the influence of e.g. local access to fruit and vegetables through grocery stores, local food policies, exposure to mass media and commercials, and fruit and vegetable availability in leisure time facilities for children and adolescents, like for instance local sport clubs.

Across the identified areas for which research is lacking, future research would benefit from improvements in design and methodology. Almost every paper identified within this review was based on cross sectional data, and the need for future longitudinal analyses of children and adolescents' fruit and vegetable consumption is evident.

Half of the papers identified by this review are based on US study populations. There is therefore an obvious lack of knowledge concerning predictors of fruit and vegetable consumption among children and adolescents from other parts of the world.

Only few papers apply a theoretical approach. Introducing more theory based research may lead to more systematic research designs that ensure sound analytical models with sufficient confounder control. In addition, the theoretical frameworks (if any) of the papers included in the present review are mostly psychosocial and do not consider more structural environmental influences like nutritional policies and availability of fruit and vegetable in the different settings that children and adolescents take part in.

We strongly recommend that future studies keep a very broad and comprehensive theoretical scope, in order not to exclude important etiological components of importance for child and adolescent fruit and vegetable intake.

Introducing new comprehensive theoretical models should be accompanied by multilevel analytical approaches from which contextual effects can be estimated [127]. To date, hierarchical models have been applied in a number of papers to adjust for the error introduced by cluster sampling [e.g. [55,91,92,98]], but so far multilevel modelling of the effect of contextual factors has only been conducted within a few papers [24,25].

### Conclusion and recommendations for practice

Despite the lack of consistent evidence for many potential determinants of fruit and vegetable intake in children and adolescents, a few recommendations for practice can be provided based on the present review.

First of all, since fruit and vegetable intake appears to decline with age among children and adolescents, the present review confirms that intervention efforts are indeed needed to promote fruit and vegetable intake across childhood and adolescence. Furthermore, interventions to promote fruit and vegetable intake should especially be aiming at reaching youth from lower SEP groups, and specific efforts should be made to also reach boys. Such interventions should aim at improving preferences for fruits and vegetables, for example by more frequent exposure [128] to fruits and vegetables by means of taste-testing games or school fruit and vegetable schemes [129]. Such interventions can also help improve availability of fruit and vegetables in schools [42].

Despite presenting solid documentation of the influence of several factors on fruit and vegetable consumption among children and adolescents this review also reveals that a long list of factors have only been sparsely investigated, and for several areas research is totally absent. Although the quality improvement of the research on children and adolescents' fruit and vegetable intake has been pronounced during recent years, a number of methodology problems have been identified. There is a need for further internationally comparative studies. At best, these should be theory-based multi-level studies in which both personal and environmental factors (family, school, local community, and national factors) are considered within a longitudinal design although we do realise that exploring such a broad range of potential determinants comes with measurement problems (e.g. long questionnaires or single item assessment of various constructs) [130]. Such future research will generate more information on determinants and mediators of fruit and vegetable consumption among children and adolescents on which coming interventions should be tailored.

## Competing interests

The author(s) declare that they have no competing interest.

## Authors' contributions

MR directed the overall study and wrote the manuscript by incorporating critical inputs from all authors. KIK, PD and JB conceived the study. MR, RK and EB conducted the literature search and MR, RK and PD participated in the selection and analysis of included papers. All authors contributed to the design of the analysis. All authors read and approved the final manuscript.

## Supplementary Material

Additional File 1 The additional file includes the large summary table 5. This comprehensive summary table contains detailed information on design, analysis, and results of all papers included in the present review. It will be made available at Click here for file
