# Supplementary material for: Determinants of fruit and vegetable consumption among children and adolescents: a review of the literature. Part I: quantitative studies
Source: Int J Behav Nutr Phys Act. 2006 Aug 11;3:22. doi: 10.1186/1479-5868-3-22 (PMC1564033; doi:10.1186/1479-5868-3-22)
Supplement: Additional File 1 — The additional file includes the large summary table 5. This comprehensive summary table contains detailed information on design, analysis, and results of all papers included in the present review. It will be made available at [file 1479-5868-3-22-S1.doc]

**Table 5: Summary table / part I**

| **Reference no.** | **Country** | **Population** | **Design** | **Theoretical basis** | **Instrument** | **Statistical analysis** |
| --- | --- | --- | --- | --- | --- | --- |
|  |  |  |  |  |  |  |
| **(17) Musaiger & Gregory,**  **J Roy Soc Health,**  **1992;112(4):159-162** | Bahrain | 1,673 students  Age: 6-22 | Cross sectional interview survey | No theory applied | 24-h recall | Descriptive analysis |
| **(18) Ahmed et al.,**  **Public Health Nutr,**  **1998;1(2):83-92** | Bangladesh | 384 girls  Age: 10-16 | Cross sectional questionnaire survey | No theory applied | FFQ | One-way analysis of variance |
| **(19) Shi et al.,**  **Eur J Clin Nutr,**  **2005;59(12):1439-1448** | China | 824 students  Age: 12-14  Junior level 1, 2 | Cross sectional questionnaire survey | No theory applied | FFQ | Multiple linear regression analysis |
| **(20) Wang et al.,**  **J Nutr,**  **2002;132:430-438** | China | 984 children  Age: 6-13 | Longitudinal questionnaire survey | No theory applied | 24-h recall | Multiple logistic regression analysis |
| **(21) Vijayapushpam et al.,**  **Publ Health Nutr,**  **2003;6(7):683-688** | India | 272 students  Age: 12–14  Class: 8 | Cross sectional questionnaire survey | No theory applied | FFQ | Chi-square test |
| **(22) De Bourdeaudhuij & Oost,**  **Psychol Health,**  **2000;15:751-770** | Belgium | 208 child/parent pairs  Mean age: 15, range: 12-18 | Cross sectional  questionnaire survey | Social Learning Theory / Theory of Planned Behaviour | FFQ / subjective measure | Multiple hierarchical regression analysis |
| **(23) Vereecken et al.,**  **J Adolesc Health,**  **2004;34:330-338** | Belgium | 12,490 students  Age: 11-24  Grade: 1-6 | Cross sectional questionnaire survey | No theory applied | FFQ | Multiple logistic regression analysis |
| **(24) Vereecken et al.,**  **Eur J Clin Nutr.**  **2005a;59(2):271-277** | Belgium-Flanders | 12,360 students/157 school principals  Age: 11-18  Primary school: Grade: 5-6  Secondary school : Grade: 1-6 | Cross sectional questionnaire survey | No theory applied | FFQ | Multilevel logistic regression analysis |
| **(25) Vereecken et al.,**  **Eur J Public Health,**  **2005b;15(3):224-232** | 28 European countries | 114,558 students  Age: 11, 13, 15 | Cross sectional questionnaire survey | No theory applied | FFQ | Multilevel logistic regression analysis |
| **(26) Osler & Hansen,**  **Scand J Soc Med,**  **1993;21(2):135-140** | Denmark | 674 students  Mean age: 12.5, 14.5  Class: 6, 8 | Cross sectional questionnaire survey | No theory applied | FFQ | Descriptive analysis |
| **(27) Adamson et al.,**  **J Hum Nutr Diet,**  **1992;5:371-385** | England | 379 students  Age: 11-12 | Cross sectional questionnaire survey | No theory applied | Food diary | Descriptive analysis |
| **(28) Cartwright et al.,**  **Health Psychol,**  **2003;22(4):362-369** | England | 4,320 students  Mean age: 11.8, SD=0.35  Grade: 7 | Cross sectional questionnaire survey | No theory applied | FFQ | Multiple logistic regression analysis |
| **(29) Crawley & While,**  **J Epidemiol Community Health,**  **1996;50:306-312** | England | 2,957 adolescents  Age: 16-17 | Cross sectional questionnaire / interview survey | No theory applied | Food diary | Log-linear models, modelling by Poisson distribution and identity link |
| **(30) Glynn et al.,**  **J Hum Nutr Diet,**  **2005;18:7-19** | England | 1,528 children  Age: 7.5, SD=0.12 | Cross sectional questionnaire survey | No theory applied | Food diary | Mann-Whitney U-test |
| **(31) New & Livingstone,**  **Public Health Nutr,**  **2003;6(5):497-504** | England | 504 students  Mean age: 12.2-15.2  Year: 7-10 | Cross sectional questionnaire survey | No theory applied | Information not provided | Mann-Whitney U test |
| **(32) Wardle et al.,**  **Prev Med,**  **2003;36:721-730** | England | 4,320 students  Mean age: 11.8 | Cross sectional questionnaire survey | No theory applied | FFQ | Chi-square test, multiple logistic regression analysis |
| **(33) Haapalahti et al.,**  **Public Health Nutr,**  **2003;6(4):365-370** | Finland | 404 children  Age: 10-11 | Cross sectional questionnaire survey | No theory applied | FFQ | Chi-square test |
| **(34) Laitinen et al.,**  **Scand J Soc Med,**  **1995;23(2):88-94** | Finland | 691 children/adolescents  Age: 9, 12, 15 | Cross sectional questionnaire survey | No theory applied | 48-h recall | Kruskal-Wallis one-way analysis of variance |
| **(35) Roos et al.,**  **Prev Med,**  **2001;33:282-291** | Finland | 76,201 students  Mean age: 15.3, SD=0.62 | Cross sectional questionnaire survey | No theory applied | FFQ | Multiple logistic regression analysis |
| **(36) Yannakoulia et al.,**  **Eur J Clin Nutr,**  **2004;58:580-586** | Greece | 4,299 students  Age: 11.5, 13.5, 15.5  Primary education: Grade: 6  Secondary education: Grade: 2,4 | Cross sectional questionnaire survey | No theory applied | FFQ | Descriptive analysis, chi square test, analysis of variance |
| **(37) Paulus et al.,**  **Eur J Clin Nutr,**  **2001;55:130-136** | Luxembourg | 1,526 students  Age: 12-17 | Cross sectional interview survey | No theory applied | FFQ | Chi-square test |
| **(38) Kremers et al.,**  **Appetite,**  **2003;41:43-50** | Netherlands | 1,771 students  Mean age: 16.5, SD = 0.8 | Cross sectional questionnaire survey | No theory applied | FFQ + perceived intake | The Tukey-HSD method |
| **(39) Van Lenthe et al.,**  **Eur J Public Health,**  **2001;11:43-50** | Northern Ireland | Baseline: 509 students  Age: 12  Follow up: 455 students  Age: 15 | Cross sectional / longitudinal questionnaire survey | No theory applied | Food record | t-test, generalised estimated equations |
| **(40) Warwick et al.,**  **Nutrition & Food Science,**  **1999;5:229-236** | Northern Ireland | 764 students / 516 parents  Age: 9-17 | Cross sectional questionnaire survey | No theory applied | FFQ | Chi-square test |
| **(41) Bere & Klepp,**  **Public Health Nutr,**  **2004;7(8):991-998** | Norway | 1,950 students / 1,647 parents  Mean age: 11.8  Grade: 6, 7 | Cross sectional questionnaire survey | Social Cognitive Theory | FFQ | Multiple linear regression analysis |
| **(42) Bere & Klepp,**  **Int J Behav Nutr Phys Act, 2005;2:15** | Norway | 816 students  Mean age (baseline): 11.8  Grade (baseline): 6, 7 | Longitudinal questionnaire survey | Social Cognitive Theory | FFQ | Multiple linear regression analysis |
| **(43) Lien et al.,**  **Public Health Nutr,**  **2002a;5(5):671-681** | Norway | 613 students  Age: 15 | Cross sectional questionnaire survey | Social Cognitive Theory / Problem Behaviour Theory | FFQ | Multiple linear regression analysis |
| **(44) Inchley et al.,**  **J Hum Nutr Diet,**  **2001;14:207-216** | Scotland | 14,669 students  Mean age 11.5, 13.5, 15.5  Primary 7, secondary 2, secondary 4 | Cross sectional questionnaire survey | No theory applied | FFQ | Chi-square test, multiple logistic regression analysis, Mantel Haenszel test |
| **(45) Longbottom et al.,**  **J Hum Nutr Diet,**  **2002;15:271-279** | Scotland | 36 children  Mean age: 6.5, SD = 1.0 | Cross sectional interview survey | No theory applied | Food record | Spearman’s rank correlation coefficient test |
| **(46) Sweeting et al.,**  **Eur J Clin Nutr,**  **1994;48:736-748** | Scotland | 908 adolescents  Age: 15, 18 | Cross sectional /longitudinal survey. Interviews / questionnaires | No theory applied | FFQ | Chi-square test, Mann-Whitney, Kruskal-Wallis |
| **(47) Wrieden W,**  **Health Educ J,**  **1996;55:185-194** | Scotland | 1,880 students  Age: 10-11  Final year at primary school | Cross sectional questionnaire survey | No theory applied | FFQ / 24-h recall | Chi-square test |
| **(48) Höglund et al.,**  **Eur J Clin Nutr,**  **1998;52:784-789** | Sweden | 7,605 students  Age: 14-15  Grade: 8 | Cross sectional questionnaire/interview survey | No theory applied | FFQ | Multiple logistic regression analysis, Pearson’s correlation coefficients |
| **(49) Samuelson et al.,**  **Am J Clin Nutr,**  **1971;24:1361-1373** | Sweden | 1,401 children  Age: 4, 8, 13 | Cross sectional interview survey | No theory applied | FFQ | Multiple linear regression analysis |
| **(50) Samuelson et al.,**  **Acta Pædiatr,**  **1996; Suppl 415:1-20** | Sweden | 411 adolescents  Age: 15 | Cross sectional questionnaire survey | No theory applied | FFQ /  Food record | Student’s t-test, multiple regression analysis |
| **(51) Sjöberg et al.,**  **Eur J Clin Nutr,**  **2003:57;1569-1578** | Sweden | 1,245 students  Mean age: 15.5, range: 15-16  Grade 9 | Cross sectional questionnaire / interview survey | No theory applied | Record of habitual diet history | One-way analysis of variance |
| **(52) Von Post-Skagegård et al.,**  **Eur J Clin Nutr,**  **2002;56:532-538** | Sweden | Baseline: 411 adolescents  Age: 15,  Follow up 1: 338 adolescents Age: 17,  Follow up 2: 208 adolescents Age: 21 | Longitudinal interview survey | No theory applied | FFQ | Multi-factorial analysis of variance |
| **(53) George & Krondl,**  **Nutr Behav,**  **1983;1:115-125** | Canada | 135 students  Age: 14-17  Grade: 10 | Cross sectional questionnaire survey | No theory applied | FFQ | Kendall’s tau correlations |
| **(54) Myres & Kroetsch,**  **Can J Public Health,**  **1978;69:208-221** | Canada | 4,227 children/adolescents  Age: 5-19 | Cross sectional survey. Information on instrument not provided | No theory applied | Information not provided | Descriptive analysis |
| **(55) Baranowski et al.,**  **J Am Coll Nutr,**  **1997;16(3):216-223** | USA | 2,924 students  Age: 7-13  Grade: 3 | Cross sectional questionnaire survey | No theory applied | Food record | Mixed linear model regression analysis |
| **(56) Baxter & Thompson,**  **J Nutr Educ Behav,**  **2002;34:166-171** | USA | 237 students  Grade: 4 | Cross sectional interview / observational survey | No theory applied | Same/next day school lunch recall | Mixed model analysis of variance |
| **(57) Beech et al.,**  **J Adolesc Health,**  **1999;24:244-250** | USA | 2,213 students  Grade: 9 | Cross sectional questionnaire survey | No theory applied | FFQ | Chi-square test |
|  |  |  |  |  |  |  |
|  |  |  |  |  |  |  |
|  |  |  |  |  |  |  |
| **(58) Bowman et al.,**  **Pediatrics,**  **2004;113(1):112-118** | USA | 6,212 children/adolescents  Age: 4-19 | Cross sectional interview survey | No theory applied | 24-h recall | Multiple linear regression analysis |
| **(59) Boynton-Jarrett et al., Pediatrics,**  **2003;112(6):1321-1326** | USA | 548 students  Baseline:  Mean age: 11.7, SD=0.75, Grade: 6, 7  Follow up:  Mean age: 13.25, SD=0.78 | Longitudinal questionnaire survey | No theory applied | FFQ | Multiple (controlling for clustered sample design) linear regression analysis |
| **(60) Burdine et al.,**  **J Sch Health,**  **1984;54(2):87-90** | USA | 2,695 students  Grade: 7, 8 | Cross sectional questionnaire survey | No theory applied | FFQ | Chi-square test |
| **(61) Campbell et al.,**  **Health Educ Behav,**  **1999;26(4):513-534** | USA | 2,213 students  Mean age: 14.8, SD=0.5  Grade: 9 | Cross sectional questionnaire survey | The Stages-of-Change Trans-theoretical Model | FFQ | Mixed model analysis of variance |
|  |  |  |  |  |  |  |
|  |  |  |  |  |  |  |
| **(62) Cross et al.,**  **J Am Diet Assoc,**  **1994;94(12):1398-1403** | USA | 290 students  Grade: 5, 6 | Cross sectional questionnaire survey | No theory applied | Type of food item eaten for snack | Descriptive analysis |
| **(63) Cullen et al.,**  **J Am Diet Assoc,**  **2000;100(12): 1482-1486** | USA | 594 students  Grade: 4, 5 | Cross sectional questionnaire survey | No theory applied | Lunch food record | Pearson’s correlation coefficients, analysis of variance / covariance |
| **(64) Cullen et al.,**  **Health Educ Behav,**  **2003;30(5):615-626** | USA | 287 students / 88 parents  Grade: 4-6 | Cross sectional questionnaire survey | Social Cognitive Theory | Food record | Structural equation modelling |
| **(65) Cullen & Zakari,**  **Am J Public Health,**  **2004;94(3):463-467** | USA | 540 students  (two cohorts: 283/257)  Age: 9-11 (baseline)  Grade: 4, 5 | Longitudinal questionnaire study | No theory applied | Lunch food record | Wilcoxon signed rank tests |
| **(66) Cullen et al.,**  **Obes Res,**  **2004;suppl 12:S20-S31** | USA | 114 girl student/parent pairs  Age: 8-10 | Cross sectional questionnaire survey | No theory applied | 24-h recall | Hierarchical stepwise linear block regression analysis |
|  |  |  |  |  |  |  |
| **(67) Domel et al.,**  **Health Educ Res,**  **1996;11(3):299-308** | USA | 252 students  Grade: 4, 5 | Cross sectional questionnaire survey | Social Cognitive Theory | Food record | Multiple linear regression analysis |
| **(68) French et al.,**  **Int J Obes,**  **2001;25:1823-1833** | USA | 4,746 students  Mean age: 14.9, range: 11-18  Grade: 7-12 | Cross sectional questionnaire survey | No theory applied | FFQ | Mixed model linear regression analysis |
| **(69) Fulkerson et al.,**  **Prev Med,**  **2004;38:865-875** | USA | 4,734 students  Mean age/boys: 14.9, SD=1.7 Mean age/girls: 14.7, SD=1.7 | Cross sectional questionnaire survey | No theory applied | FFQ | Multiple linear model analysis |
|  |  |  |  |  |  |  |
| **(70) Gillman et al.,**  **Arch Fam Med,**  **2000;9:235-240** | USA | 16,202 children  Age: 9-14 | Cross sectional questionnaire survey | No theory applied | FFQ | Multiple logistic regression analysis |
|  |  |  |  |  |  |  |
|  |  |  |  |  |  |  |
| **(71) Greger et al.,**  **Ecol Food Nutr,**  **1979;7:213-218** | USA | 178 girls  Mean age: 12.8 +/- 0.1  Grade: 6-8 | Cross sectional interview survey | No theory applied | 24-h recall | Descriptive analysis |
|  |  |  |  |  |  |  |
| **(72) Hanson et al.,**  **Public Health Nutr,**  **2005;8(1):77-85** | USA | 902 students/parents pairs  Middle and high school | Cross sectional  questionnaire survey | No theory applied | FFQ | General linear modelling (adjusted),  t-tests of trend |
|  |  |  |  |  |  |  |
| **(73) Hearn et al.**  **J Health Educ,**  **1998;29(1): 26-32** | USA | Sample I: Grade 3 students and parents from 16 schools (individual level sample size not reported). Family survey: 13 randomly selected families from sample I  Sample II: Students from 45 elementary schools (individual level sample size not reported) | Cross-sectional questionnaire survey.  Family survey based on telephone interview | Social Cognitive Theory | Food record | Multivariate mixed model linear regression analysis, Pearson’s correlation coefficients |
| **(74) Heunemann et al.,**  **J Am Diet Assoc,**  **1968;53:17-24** | USA | 122 students  Age: 16-17  Grade: 11, 12 | Cross sectional questionnaire survey | No theory applied | Food diaries | Not described |
|  |  |  |  |  |  |  |
|  |  |  |  |  |  |  |
| **(75) Kratt et al.,**  **Health Educ Behav,**  **2000;27(4):471-482** | USA | 1,196 child/parent pairs  Grade: 4 | Cross sectional questionnaire / interview survey | Social Cognitive Theory | 24-h recall | Analysis of variance, multi group structural equation modelling |
|  |  |  |  |  |  |  |
| **(76) Kubik et al.,**  **Am J Public Health,**  **2003;93(7):1168-1173** | USA | 598 students  Grade: 7 | Cross sectional interview / observational survey | No theory applied | 24-h-recall | Multivariate general linear mixed modelling |
|  |  |  |  |  |  |  |
| **(77) Lantz & Wood,**  **J Am Diet Assoc,**  **1958;34:1199-1207.** | USA | 365 students  Age: 6-16 | Cross sectional questionnaire survey | No theory applied | Food record | No statistical test |
| **(78) Levine & Gurhrie,**  **Fam Econ Nutr Rev,**  **1997;10(3):20-35** | USA | 1,087 adolescents  Age: 13-18 | Cross sectional interview survey | No theory applied | FFQ | Chi-square test, student t-test |
|  |  |  |  |  |  |  |
| **(79) Lien et al.,**  **Am J Health Promot,**  **2002b;16(4):189-197** | USA | 1,406 students  Grade: 7, 8 | Longitudinal questionnaire survey | Theory of Planned Behaviour | FFQ | Path analysis |
|  |  |  |  |  |  |  |
| **(80) Lowry et al.,**  **JAMA, 1996;276(10):792-797** | USA | 6,321 adolescents  Mean age: 14.5, range 12-17 | Cross sectional interview survey | No theory applied | FFQ | Multiple logistic regression analysis |
| **(81) Lowry et al.,**  **J Sch Health;**  **2002;72(10):413-421** | USA | 15,349 students  Grade: 9-12 | Cross sectional questionnaire survey | No theory applied | FFQ | Multivariate logistic regression analysis |
| **(82) Lytle et al.,**  **J Nutr Educ Behav,**  **2003;35:170-178** | USA | 3,878 students  Mean age: 12.8, SD = 0.38  Grade: 7 | Cross sectional questionnaire survey | Social Cognitive Theory / Theory of Planned Behaviour | FFQ | Generalised linear mixed model (event rate ratios (ERR) for predictor variable (10^th^, 25^th^, 75^th^, and 90^th^ percentiles), reference = median) |
| **(83) Matheson et al.,**  **Am J Clin Nutr,**  **2004;79:1088-1094** | USA | Sample 1: 91 students  Mean age: 8.6, range: 7.8-9.6 Grade: 3  Sample 2: 129 students  Mean age: 9.6, range: 9.0-11.5 Grade: 5 | Cross sectional interview survey | No theory applied | 24-h recall | Two-factor analysis of variance |
| **(84) Melnik et al.,**  **J Am Diet Assoc,**  **1998;98:159-164** | USA | 1,397 students  Grade: 2, 5 | Cross sectional  questionnaire survey | No theory applied | FFQ | Least square mean statistics |
|  |  |  |  |  |  |  |
|  |  |  |  |  |  |  |
|  |  |  |  |  |  |  |
|  |  |  |  |  |  |  |
| **(85) Neumark-Sztainer et al.,**  **Prev Med,**  **1996;25:497-505** | USA | 36,284 students  Mean age: 15.0, SD=1.8  Grade: 7-12 | Cross sectional questionnaire survey | No theory applied | FFQ | Multiple logistic regression analysis |
| **(86) Neumark-Sztainer et al.,**  **Prev Med,**  **2003a;37:198-208** | USA | 3,957 students  Mean age: 14.9, SD=1.7 | Cross sectional questionnaire survey | Social Cognitive Theory | FFQ | Structural equation modelling |
| **(87) Neumark-Sztainer et al.**  **J Am Diet Assoc,**  **2003b;103:317-322** | USA | 4,746 students  Mean age: 14.9, range: 11-18  Grade: 7-12 | Cross sectional  questionnaire survey | No theory applied | FFQ | Multiple linear regression analysis, test for linear trend |
|  |  |  |  |  |  |  |
| **(88) Nicklas et al.,**  **Sch Food Serv Res Rev,**  **1993;17(2):125-132** | USA | 393 children  Age: 10 | Cross sectional interview survey | No theory applied | 24-h recall | Descriptive analysis |
|  |  |  |  |  |  |  |
|  |  |  |  |  |  |  |
|  |  |  |  |  |  |  |
| **(89) Pearman et al.,**  **Am J Health Behav,**  **2000;24(3):220-28** | USA | 6,627 students  Grade: 9-12 | Cross sectional questionnaire survey | No theory applied | Eating FV on the previous day (yes/no) | Chi-square test |
| **(90) Rafiroiu et al.,**  **Am J Health Behav,**  **2002;26(3):200-212** | USA | 2,021 students  Grade: 8, 11 | Cross sectional interview survey | Social Cognitive Theory | Responses to questions on compliance with recommen-dations | Chi-square test |
| **(91) Resnicow et al.,**  **Health Psychol,**  **1997;16:272-276** | USA | 1,398 students  Mean age: 8.7, range: 7-11  Grade: 3 | Cross sectional questionnaire survey | Social Cognitive Theory | Food diary | Mixed linear model regression analysis |
| **(92) Reynolds et al.,**  **J Am Coll Nutr,**  **1999a;18(3):248-254** | USA | *Alabama*: 1,169 students,  Age: 8-10 / Grade: 4  *Georgia*: 1,481 students,  Age: 8-10 / Grade: 3  *Louisiana*: 608 students,  Age: 14-16 / Grade: 9  *Minnesota*: 500 students,  Age: 9-11 | Cross sectional questionnaire / interview surveys | No theory applied | *Alabama*: 7 day 24-h recall  *Georgia:* 7 day food records  *Louisiana:* 1 day 24-h recall  *Minnesota:* 3-4 day 24-h recall | Mixed linear regression model |
| **(93) Reynolds et al.,**  **J Nutr Educ,**  **1999b;31:23-30** | USA | 414 students  Grade: 3 | Cross sectional questionnaire / interview survey | Social Cognitive Theory | 24-h recall | Structural equation modelling |
|  |  |  |  |  |  |  |
|  |  |  |  |  |  |  |
|  |  |  |  |  |  |  |
|  |  |  |  |  |  |  |
|  |  |  |  |  |  |  |
| **(94) Siega-Riz et al.,**  **J Adolesc Health,**  **1998;22:29-36** | USA | 1,310 adolescents  Age: 11-18 | Cross sectional questionnaire survey | No theory applied | 24 h recall / food record | Univariate linear regression analysis |
|  |  |  |  |  |  |  |

| **(95) Skinner et al.,**  **J Nutr Educ,**  **1984;16(4):164-167** | USA | 148 students  Mean age = 17.2, range: 16-18 | Cross sectional questionnaire survey | No theory applied | Food record | Chi-square test |
| --- | --- | --- | --- | --- | --- | --- |
|  |  |  |  |  |  |  |
| **(96) Taveras et al.,**  **Pediatrics,**  **2005;116(4):e518-e534** | USA | 14,355 children/adolescents  Age: 9-14 | Cross sectional | No theory applied | FFQ | Multivariate linear regression analysis |
| **(97) Utter et al.,**  **J Am Diet Assoc,**  **2003;103:1298-1305** | USA | 4,746 students  Mean age: 14.9 | Cross sectional questionnaire survey | No theory applied | FFQ | Multivariate linear regression analysis |
|  |  |  |  |  |  |  |
|  |  |  |  |  |  |  |
|  |  |  |  |  |  |  |
|  |  |  |  |  |  |  |
| **(98) Videon & Manning,**  **J Adolesc Health,**  **2003;32:365-373** | USA | 18,177 students  Mean age: 15.9, range: 11-21 | Cross sectional interviews survey | No theory applied | FFQ | Multiple (controlling for clustered sample design) logistic regression analysis |
|  |  |  |  |  |  |  |
|  |  |  |  |  |  |  |
|  |  |  |  |  |  |  |
|  |  |  |  |  |  |  |
|  |  |  |  |  |  |  |
|  |  |  |  |  |  |  |
| **(99) Wolfe & Campbell,**  **J Am Diet Assoc,**  **1993;93:1280-1284** | USA | 1,615 students  Mean age: 7.9 / Grade: 2  Mean age: 10.9 / Grade: 5 | Cross sectional questionnaire survey | No theory applied | 24-h recall | Chi-square test, multiple logistic regression analysis |
| **(100) Xie et al.,**  **Prev Med,**  **2003;36:30-40** | USA | 3,201 students  Mean age: 14.6, range: 11-20 | Cross sectional questionnaire survey | No theory applied | FFQ | Analysis of covariance |
| **(101) Young & Fors,**  **J Sch Health,**  **2001;71(10):483-488** | USA | 3,155 students  Grade: 9-12 | Cross sectional questionnaire survey |  | FFQ | Chi-square, Kendall’s tau b, one-way ANOVA |
| **(102) Young et al.,**  **J Nutr Educ Behav,**  **2004;36:2-12** | USA | 366 students  Mean age: 12.9 / Grade: 6  Mean age: 13.8 / Grade: 7  Mean age: 14.7 / Grade 8 |  | Social Cognitive Theory | FFQ | Hierarchical linear regression analysis |
| **(103) Bell & Swinburn,**  **Eur J Clin Nutr,**  **2004;58:258-263** | Australia | 1,656 students  Age: 5-15 | Cross sectional interview survey |  | 24-h recall | Paired t-test, multiple linear regression analysis |
| **(104) Giskes et al.,**  **Public Health Nutr,**  **2002;5(5):663-669** | Australia | 654 adolescents  Age 13-17 | Cross sectional interview survey | No theory applied | 24-h recall | Multiple logistic regression analysis, general linear models |
| **(105) Milligan et al.,**  **Aust N Z J Public Health,**  **1998;22(4):485-493** | Australia | 504 students  Mean age: 18.0, 95%CI 18.0-18.1 | Cross sectional questionnaire survey | No theory applied | 24-h recall | Analysis of variance / Mann-Whitney test |
| **(106) Nowak & Speare,**  **J Paediatr Child Health,**  **1996;32:424-427** | Australia | 791 students  Mean age: 12.9, range: 12-15  Year: 8 | Cross sectional questionnaire survey | No theory applied | FFQ | Regression analysis |
| **(107) Harding et al.,**  **J NZ Diet Assoc,**  **1988;42(1):4-13** | New Zealand | 145 students  Mean age: 15.0 +/-0.4  4^th^ form | Cross sectional interview survey | No theory applied | 24-hour recall | Descriptive analysis |
| **(108) Worsley et al.,**  **J Paediatr Child Health,**  **1993;29:209-214** | New Zealand | 667 adolescents  Age: 15 | Cross sectional questionnaire/interview survey | No theory applied | FFQ | Contingency table analysis, analysis of variance, chi-square tests |
| **(109) Williams et al.,**  **Aust J of Nutr Dietet,**  **1993;50(4):156-163** | Tasmania | 2,082 students  Age: 12-15  Grade: 7-10 | Cross sectional questionnaire survey | No theory applied | FFQ | Chi-square test, median test |
| **(110) Woodward,**  **Br J Nutr,**  **1985a;54:325-333** | Tasmania | 1,055 students  Age: 11-16  Grade: 7-10 | Cross sectional questionnaire survey | No theory applied | Food record | The median test |
| **(111) Woodward,**  **J Food Nutr,**  **1985b;42(1):7-12** | Tasmania | 1,055 students  Age: 11-16 | Cross sectional questionnaire survey | No theory applied | Food record | The median test |
| **(112) Woodward,**  **Hum Nutr Appl Nutr,**  **1886;40(3):185-194** | Tasmania | 1,055 students  Age: 11-16  Grade: 7-10 | Cross sectional questionnaire survey | No theory applied | Food record | The median test |
| **(113) Woodward et al.,**  **Appetite,**  **1996;27:109-117** | Australia | 2,082 students  Age: 12-15 | Cross sectional questionnaire survey | No theory applied | FFQ | Multiple linear regression analysis |
| **(114) Rojas,**  **Arch Latinoam Nutr,**  **2001;51(1):81-85** | Costa Rica | 274 students  Mean age: 15 +/- 1.3 | Cross sectional questionnaire survey | No theory applied | Food record | Analysis of variance |
|  |  |  |  |  |  |  |
|  |  |  |  |  |  |  |
|  |  |  |  |  |  |  |
|  |  |  |  |  |  |  |
|  |  |  |  |  |  |  |
|  |  |  |  |  |  |  |

F = fruit; V = vegetables; J = juice; FJ = fruit juice; FFQ = food frequency questionnaire; 24-h (48-h) recall = 24 hour (48 hour) recall; CI = confidence interval

**Table 5: Summary table /** part II

| **Ref. no.** | **Independent variables*** | **Results** |
| --- | --- | --- |
|  |  | **Associated variables / Effect sizes** |
|  |  |  |
| **(17)** | Age, gender, meal type (stratified) | *Lunch*: the consumption of F was almost twice the level among girls than among boys (31.9% vs. 17.1%).  *Afternoon snack*: no marked differences  *Supper*: the consumption of V was higher among boys than girls (12.4% vs. 1.3%) |
| **(18)** | Family income, expenditure on foods, father’s education, mother’s education | High level of mother’s education was associated with high frequency of F consumption (p=0.016)  Low expenditure on foods was associated with high frequency of leafy V consumption (p=0.03) |
| **(19)** | Gender, urbanisation, household SES (based on household material possessions), mother’s/father’s education, family size | *Frequency of FV consumption:*  Household SES: β=0.16, p<0.001  Gender (girls vs. boys): β=0.52, p<0.001 |
| **(20)** | Urbanisation, family income, mother’s education, region (central, coast, southwest), baseline energy intake  Confounders: age, gender, father’s occupation | Urban children were less likely to track a diet high in FV (OR=0.28 (0.13-0.63)) than rural children. Children whose mothers have higher educational levels were less likely to maintain a high FV diet (OR=0.46 (0.28-0.76)). Children living in coastal areas were more likely to track lower FV consumption (OR=0.28 (0.13-0.56)) |
| **(21)** | SES (income level) | Most frequent intake of green leafy V and F among high SES students compared to low SES students (p-value not provided) |
| **(22)** | *Children’s and parents’ reports: Personal determinant:* attitude, perceived social support, self-efficacy, modelling (perceived intake of significant others), intention  *Interactions around food:* Shopping: shopping is shared, healthy food is asked for to be brought, food asked for is actually bought  *Food routines*: eating breakfast is shared, eating hot meals is shared, meals have a special meaning  *Communication*: communication of dislike, use of positive strategies, use of negative strategies  *Rules*: obligation rules, restriction rules  *General family characteristics:*  Cohesion, adaptation, parent-child interaction | *‘Objective’* measure of intake (FFQ):  *F:* Personal determinants/ self-efficacy: β=0.33, p<0.01; friend modelling: β=0.17, p<0.05; intention: β=0.23, p<0.05. Explained variance =31%. Food interactions/ healthy food asked for: β=0.23, p<0.05; food asked for is bought: β=-0.19, p<0.05. Explained variance = 35%. *V:* Personal determinants / self-efficacy: β=0.37, p<0.01. Explained variance = 17%. Food interactions/ food asked for is brought: β=-0.22, p<0.01; use of negative strategies: β=-0.19, p<0.05. Explained variance =24%  *‘Subjective’* measure of intake (evaluation of own intake):  *F:* Personal determinants/ self-efficacy: β=0.47, p<0.001; social support: β=0.15, p<0.05; family modelling: β=0.29, p<0.001. Explained variance = 55%. Food interactions/ healthy food asked for: β=0.12, p<0.10; food asked for is bought: β=-0.12, p<0.10. Explained variance = 57%. *V:* Personal determinants/ attitudes: β=0.43, p<0.001; self-efficacy: β=0.43, p<0.001. Explained variance = 51%. Food interactions/ parent-child interaction: β=0.15, p<0.05. Explained variance =52% |
| **(23)** | Age, gender, student educational level (general, technical, vocational), parental occupation | Younger students more frequently ate F than the older students (p<0.01). Girls more frequently ate F and V than boys (p<0.01). Positive associations between student educational level (most frequent intake among student with general education, p<0.001) and parental occupation (F: p=0.007, V: p<0.001) and frequency of both F and V consumption |
| **(24)** | *Individual level (*students’ reports): gender, grade, SES (parental occupation), school food rules, availability of F and V at school, food education programmes in 2002  *School level:*  *Students’ reports:* Aggregated individual SES at the school level.  *School principals’ reports*: Presence of vending machines/school stores, availability of food items at school, school food rules, sweets and savoury snacks, previous nutrition education programmes | Girls more frequently ate F daily than boys (no test for significance)  In secondary school, the youngest students most frequently ate F daily (no test for significance)  High SES-student most frequently ate F daily (no test for significance)  For *primary schools* no significant school level variance existed (null models) for F intake  For *secondary schools* the null model showed significant variation (school level variance = 0.106, p<0.001) for F intake, however, when individual variables were taken into account no significant school level variance was left for F intake |
| **(25)** | *Individual level*: age, gender, parental occupation, family affluence scale (car ownership, computer ownership, family holidays last year, own bedroom) (FAS)  *School level:* parental occupation (aggregated measure), family affluence scale (aggregated measure)  *National level*: family affluence scale (aggregated measure), geographical region (Western European, Central and Eastern European, Northern European, and Southern European countries) | *Two level analysis (p<0.05 – 0.001):*  In 24 countries girls more frequently ate F than boys, in 24 countries younger students more frequently ate F than older students, in 15 countries parental occupation was positively associated with frequency of F intake, in 21 countries FAS score was positively associated with frequency of F intake, students living in Southern European countries more frequently ate F than students in other areas  *Multilevel analysis (OR, 95% CI):*  *Fixed effects for eating F:* Gender: reference=Boys, girls: OR=1.398 (1.363-1.434); Age: reference=11-years, 15-year-olds: OR=0.652 (0.629-0.675); Parental occupation: reference=low, high: OR=1.166 (1.126-1.208); FAS: reference=low, high: OR=1.520 (1.471-1.572); Mean school parental occupation: OR=1.078 (1.012-1.148)  *Random effects (full model):* Country-level variance: 0.108 (0.049-0.167) (a considerable amount of the initial variation at this level was reduced by geographical region). School-level variance: 0.082 (0.070-0.094) |
| **(26)** | Gender, ethnicity, dietary knowledge | Consumption of both V and fresh F did not seem to differ between boys and girls. Immigrant students ate more V and fresh F than Danish students. Higher dietary knowledge was seen among students with daily consumption of both V and fresh F compared to those with infrequent consumption |
| **(27)** | Gender, social group (occupation of the ‘head of the household’) | V were more important sources of unavailable carbohydrate for girls than boys (12.0% vs. 9.7%). The contribution from both F and V was lowest in the low social groups: V: high=11.3%, middle=11.6%, low=10.1%; F: high=5.9%, middle=5.7%, low=4.9% |
| **(28)** | Gender, ethnicity, SES (census-level data based on zip codes provided by the pupils / Townsend Index), perceived stress, weight | *Eating 5 or more FV a day:*  Ethnicity: Blacks (OR=1.19, p=0.07) more frequent ate FV than whites (reference group), and Asians (OR=1.04, p=0.76)  Stress: stressed adolescents less frequent ate FV than non-stressed adolescents (most stressed: OR=0.78, p=0.01) |
| **(29)** | Parental smoking, gender (stratified)  Confounders: social class (occupation of head of household), region | *Boys*: higher intake of salad V (p<0.001) and FJ (p<0.001) among boys with neither parents smoking compared to boys with either/both parents smoking  *Girls*: higher intake of salad V among girls with neither parents smoking compared to girls with either/both parents smoking (p<0.05) / intake of FJ was lowest when only mother smoked or both parents smoked (p<0.005) |
| **(30)** | Gender | Boys ate 27 grams of FV per MJ of energy compared to 30 grams among girls (level of significance not reported) |
| **(31)** | Frequency of confectionery consumption from vending machines | No significant association between frequency of confectionery consumption from vending machines and intake of F or V |
| **(32)** | Socioeconomic deprivation (neighbourhood-based SES index), gender (stratified)  Confounders: ethnicity, age | *Eat five or more F V/day:*  Girls: index quintiles: Least deprived OR=1.00, second quintile OR=1.02, third quintile OR=0.80, fourth quintile OR=0.73, most deprived OR=0.71 (p-value for trend = 0.037) |
| **(33)** | SES (father’s occupation), family meal patterns (regular / no regular family dinner) | Father’s occupation was associated with frequency of V consumption (entrepreneur: 21%, upper white-collar: 40%, lower white-collar: 18%, worker: 26%, other: 29%, p=0.03) |
| **(34)** | Father’s occupational status, father’s educational status, family income | Children in families whose educational level of father or income was low consumed less F (p<0.01) |
| **(35)** | Parental educational level, family structure, parents unemployed during past year, financial situation in family, liking school, school achievement, school meals, snacks during the school day, evening meal pattern at home, physical activity, smoking, alcohol drinking, parental smoking, weight perception, body weight | *Frequency of raw V consumption*  *Odds ratios (95% CI)*  *Univariate analyses: female / male*  Parental educational level: reference=basic school, university degree=2.86 (2.71-3.22)/2.95 (2.62-3.31); Family structure: reference =both parents, father alone=0.69 (0.57-0.83)/0.73 (0.58-0.92); Parents unemployed during past year: reference =neither of them, both parents=0.64 (0.57-0.71)/0.70 (0.59-0.82); Financial situation in family: reference=very good, severe problems=0.53 (0.47-0.61)/0.56 (0.46-0.69); Liking school: reference=liking school, not at all=0.44 (0.38-0.52)/0.36 (0.30-0.43); School achievement: reference=highest quartile, lowest quartile=0.30 (0.60-0.68)/0.32 (0.32-0.39); School meals: reference =eats daily, never=0.71 (0.65-0.79)/0.72 (0.63-0.82); Snacks during the school day: reference=no, yes=0.88 (0.84-0.92)/0.93 (0.87-0.99); Evening meal pattern at home: reference=daily, no meal at home=0.68 (0.64-0.73)/0.57 (0.51-0.63); Physical activity: reference=daily or more often, <1 time/week=0.37 (0.33-0.41)/0.47 (0.41-0.53); Smoking: reference=non-smoker, at least once per week=0.64 (0.61-0.68)/0.65 (0.60-0.70); Alcohol drinking: reference=never, weekly=0.74 (0.67-0.81)/0.79 (0.71-0.87); Parental smoking: reference =neither parent smoke, both parents smoke=0.59 (0.54-0.64) /0.62 (0.56-0.69); Weight perception: reference=satisfied with weight; no pattern; Body weight: reference =normal weight, no pattern  *Multivariate analysis* (controlled for all other variables)*:* female / male  Parental educational level: reference =basic school, university degree=2.15 (1.96-2.36)/2.25 (1.99-2.54)  The most important intermediate factor group was the school factors (liking school, school achievement), followed by the family factors (structure, financial situation, and unemployment status). No major differences among boys and girls – though girls tended to eat raw V more frequently than boys |
| **(36)** | TV watching, dieting, gender, age | The more hours spent in front of the TV the less frequent consumption of F (level of significance not reported)  Younger students more frequently consumed F and V compared to older students (level of significance not reported)  Girls consumed F and V more frequently than boys (level of significance not reported)  Students on a diet, compared to students that think that their weight is fine and students having intentions to diet, were more likely to report a higher daily frequency of consumption of F (p=0.04) and raw V (p<0.01) |
| **(37)** | Gender, age, student social class (classical/theoretical curriculum vs technical curriculum) | More girls than boys ate F (p<0.001) and V (p<0.001) daily  Frequency of F and V consumption decreased by increasing age (p<0.01). Students from classical/theoretically learning options more frequently consumed F (p<0.001) and V (p<0.001) than students from technical learning options |
| **(38)** | Parenting style (authoritative, authoritarian,  Indulgent, neglectful)  Confounders: gender, age, educational level of student, BMI, ethnicity, religiosity | Adolescents in authoritative home ate more F than adolescents raised with other parenting styles. Adolescents from indulgent homes ate more F than adolescents from authoritarian or neglectful homes. No difference was found between adolescents from authoritarian and neglectful homes (p<0.01) |
| **(39)** | Occupation of the main breadwinner in the family, gender (stratified) | *Cross-sectional analyses*: At age 15, boys (p=0.05) and girls (p=0.02) in the manual group consumed less F compared to subjects in the non-manual group  *Longitudinal analyses including the measurements at 12 and 15 years simultaneously*: boys (beta=-0.42, p=0.03) and girls (beta=-0.87, p=0.01) in the manual group consumed less F compared to subjects in the non-manual group |
| **(40)** | *Students’ reports*: age, gender  *Parents’ reports*: parental occupation Meal (stratified) | *Lunch:* more younger students ate a piece of F at lunch than older students (p<0.001)  *Snacking*: F was a snack choice among more younger students than older students (p<0.001)  *Socioeconomic position*: more students in higher socioeconomic groups ate F compared to students in the lower socioeconomic groups (p<0.01) |
| **(41)** | *Students’ reports*: Behavioural skills, accessibility, modelling (perceived behaviour of others), intention, preferences, self-efficacy, awareness  *Parents’ reports*: Child’s behavioural skills, parent’s intake, child’s accessibility, child’s preferences | *Frequency of FV consumption:*  *Students’ reports*: accessibility: β=0.26, p<0.01; modelling: β=0.05, p=0.03; preferences: β=0.21, p<0.01; self-efficacy: β=0.09, p<0.01; awareness: β=0.12, p<0.01  *Parent reports*: parent’s intake: β=0.10, p<0.01; child’s preferences: β=0.14, p<0.01  Explained variance: 34% |
| **(42)** | Home accessibility of FV, school accessibility (school F program), modelling (perceived behaviour of others), intention, preferences, self-efficacy, awareness | *Frequency of FV consumption:*  *Intake of FV at baseline:* accessibility at home: β=0.24, p<0.01; preferences: β=0.26, p<0.01; self-efficacy: β=0.08, p<0.05; awareness: β=0.09, p<0.01. Explained variance = 29%  *Intake of FV at follow-up:* Model a: Intake at baseline not included: accessibility at home: β=0.15, p<0.01; preferences: β=0.18, p<0.01; self-efficacy: β=0.11, p<0.01; awareness: β=0.09, p<0.05. Explained variance = 17%. Intake at baseline included: only significant variable = intake at baseline: β=0.47, p<0.01. Explained variance = 33%. Model b: change in accessibility at home: β=0.14, p<0.01; change in accessibility at school: β=0.17, p<0.01; change in preferences: β=0.17, p<0.01; change in awareness: β=0.08, p<0.01; past intake: β=0.59, p<0.01. Explained variance = 43%  *Change in intake of FV*: change in accessibility at home: β=0.21, p<0.01; change in accessibility at school: β=0.16, p<0.01; change in preferences: β=0.21, p<0.01; change in awareness: β=0.07, p<0.05). Explained variance = 15% |
| **(43)** | Gender, SES (parental education)  *Personal domain*: self-evaluation, subjective health complaints, evaluation of own health, evaluation of own diet, physical activity, smoking, dieting , use of dietary supplements, meal frequency  *Family domain*: feeling monitored by parents, positive with parents, perceived parental evaluation of his/her diet, parental physical activity, parental smoking  *Friend related variables*: peer relations, best friend physically active, best friend smoking  *School/society domain:* liking school, academic achievement, antisocial behaviour | *Frequency of FV consumption:*  Evaluation of own diet: β=0.16, p<0.001; physical activity: β=0.13, p=0.001; meal frequency: β=0.11, p=0.01; relations with parents: β=0.10, p=0.02; relations with friends: β=0.11, p=0.009; gender (highest intake among girls): β=0.09, p=0.03; SES (highest intake among high SES students): β=0.09, p=0.03  Stratified according to SES and gender:  *Low SES boys*: evaluation of own diet: β=0.22, p=0.003; physical activity: β=0.17, p=0.02  *High SES boys*: physical activity: β=0.17, p=0.04  *Low SES girls*: evaluation of own diet: β=0.23, p=0.007; relations with parents (β=0.21, p=0.008)  The cross domain model explained 3.5-14.5% of the variance in the FV score varying between gender and SES |
| **(44)** | Family affluence scale (presence of telephone in home, car ownership, own bedroom), gender  For standardisation: age, school type (state / independent) | *Gender differences in pct. consuming foods daily:*  F: girls consumed more frequently than boys (1990, 1994, 1999 p<0.001); Raw V & salads: girls consumed more frequently than boys (1994, 1999 p<0.001); Cooked V: girls consumed more frequently than boys (1994 p<0.05, 1999 p<0.001)  *Associations with family affluence scale:*  Higher scores on the family affluence scale were associated with more frequent consumption: F (p<0.05); raw V and salads (p<0.001); cooked V (p<0.001) |
| **(45)** | Mother’s intake of F and V | A positive correlation between children’s and mothers’ intake was found for median densities of F (r=0.735, p<0.001) |
| **(46)** | Father’s current or previous occupation, own labour market attachment (age 18), place of residence, gender | *Cross sectional analyses:* At age 18, female ate raw V (p<0.001) and F (p<0.001) more frequently than males, adolescents from non-manual backgrounds ate V (p<0.001), F (p<0.001), and FJ (p<0.001) more frequently than adolescents from lower SES backgrounds, adolescents under education ate V (p<0.001), F (p<0.001), and FJ (p<0.001) more frequently than unemployed adolescents, adolescents living outside family ate F (p<0.05), and FJ (p<0.05) more frequently than adolescents living at home  *Longitudinal analyses*: unemployed adolescents had a higher reduction in F intake from age 12 to 18 than working adolescent or adolescents under education (p<0.05) |
| **(47)** | Gender, area (urban/rural), catchment of school (local authority rent/owner-occupier/mixed) | *Overall V consumption*: More students from owner-occupied and mixed catments than from local authority housing-scheme areas frequently consumed V (p<0.05)  *Specific F and V items:* Gender: More boys than girls frequently consumed orange J. More girls than boys frequently consumed apples and green V (p<0.05). Area: More students from rural areas than from urban areas frequently consumed apples, green V, and potatoes (p<0.05). School catchment: More students from owner-occupied catchments than from other types of catchments frequently consumed carrots, green V, and potatoes (p<0.05) |
| **(48)** | Gender, knowledge, physical condition, smoking, socioeconomic index (based on school catchment areas) | *Frequency of V consumption (p<0.001):*  Gender: girls OR=1.60; Knowledge: knowing how to eat OR=1.41; Physical condition: being fit OR=1.19; Smoking behaviour: non-smoking OR=0.58  *Frequency of F consumption (p<0.001):*  Knowledge: knowing how to eat OR=1.75; Smoking behaviour: non-smoking OR = 0.52  *Ecological analysis*: Boys from areas with higher socioeconomic status most often consumed V (r = 0.49, p<0.01) |
| **(49)** | Parental education, income per capita, area (urban/rural) (stratified), age (stratified)  Confounder: gender | *Frequency of F and V consumption:*  *Urban areas:* 4-year-olds: consumption of V was positively associated with parental education (p<0.01), F consumption was positively associated with income per capita (p<0.01) / 13-year-olds: consumption of V and berries was positively associated with parental education (p<0.001), and F consumption was positively associated with income per capita (p<0.001)  *Rural areas:* 8-year-olds: F consumption was positively associated with parental education (p<0.01) and income per capita (p<0.001) |
| **(50)** | Gender, mother’s/father’s education, mother’s/ father’s present occupation, number of children in the family, type of dwelling, smoking | Girls consumed F (p<0.01) and V (p<0.01) more frequently than boys.  Adolescents whose mothers had a high educational level more frequently consumed V than adolescents whose mothers had a lower educational level (p<0.01)  Being a smoker was associated with infrequent consumption of V (p<0.01) and F (p<0.01) |
| **(51)** | Meal pattern (regular / irregular) | Analysis only possible for girls: for both V (p<0.001) and F/berries (p=0.041) the highest intake was observed among adolescent girls with regular meal patterns |
| **(52)** | Gender, age, region (Uppsala/Trollhättan), parental educational level, BMI | From age 17 to 21, the frequency of F intake increased more among girls than boys (p<0.01)  Both boys and girls increased their frequency of intake of V from age 15 to 21 (p<0.001)  From age 17 to 21, high educational level of mother was associated with a higher increase in frequency of V intake than low educational level of mother (p<0.05) |
| **(53)** | Gender, perceived body image (stratified) | Girls more frequent than boys ate: celery (p<0.001), carrots (p<0.01), turnips (p<0.01), lettuce (p<0.01), apples (p<0.01).  *Analysis stratified by perceived body image*: stronger associations were seen for lettuce, carrots, and celery among girls who perceived themselves to have normal body images. For this group a weaker association was seen for turnips |
| **(54)** | Income index (family income / family size), gender (stratified) | The consumption of F was lower among 12-19 year old boys and girls from families with low income index. For both boys and girls V consumption varied greatly between income groups |
| **(55)** | Individual level variables:  *Students’ reports*: Day and meal of FV consumption  *Parents’ reports*: Gender, ethnicity  School level variables:  *Information from school system:* No. of students in school, annual out migration rate, pct. of students receiving free or reduced price lunch, pct. of students participating in the school lunch  *Students’ reports:* Pct. European-American children, pct. guardians with a college degree or more | Weekday lunch consumption accounted for most of the weekday servings of FV (p<0.001). Dinner was also a major contributor to V intake  Girls ate more FV than boys (p<0.001)  Pct. of European-American students at school was positively associated with total FV consumption (r=0.39)  Pct. of parents with some or more college education was positively associated with weekend consumption FV (no estimates reported)  Students from schools with greater participation rates in school lunch ate more FV at weekday lunch (r=0.49) but less FV at weekend lunch (r=-0.52)  Variance attributable to school was almost negligible (ICC:0.001-0.125) |
| **(56)** | Preferences, interview day (random effect) | High preferences were associated with high intake of F (p<0.0001) and V (p<0.0001) |
| **(57)** | Gender, ethnicity | Ethnicity: White adolescent ate FV more frequent than African-American (p<0.05) |
| **(58)** | Eating fast food  Confounders: age, gender, race/ethnicity, number of people living in the household, urbanisation, geographical region (Northeast, Midwest, South, or West) | *Between-subject comparisons*: Fast food consumption was inversely associated with consumption of F and non-starchy V (p<0.0001)  *Within-subject comparisons:* children who ate fast food on one but not both of the survey days consumed less F and non-starchy V on the day of eating fast food compared to the day of not eating fast food (p<0.0001) |
| **(59)** | Television viewing including/excluding time spent on video and computer games  Confounders: Baseline energy-adjusted F and V intake, total energy intake, age, gender, ethnicity, BMI, physical activity, frequency of sit-down dinner | *Baseline TV viewing and frequency of F and V intake (servings/day) at follow up:*  Change in intake per 1 hour increase in television viewing at baseline:  Including video/computer games: FV: -0.16 (95%CI: -0.22 - -0.10; p=0.008); F: -0.074 (95%CI: -0.08 - -0.06; p=0.0004)  Excluding video/computer games: FV: -0.19 (95%CI:­ -0.19 - -0.03; p=0.05)  *Change in TV viewing from baseline to follow up and frequency of F and V intake (servings/day) at follow up:*  Change in intake per 1 hour increase in television viewing between baseline and follow up: Including video/computer games: FV: -0.14 (95%CI: -0.22 - -0.07; p=0.025); F: -0.053 (95%CI: -0.09 - -0.01; p=0.06). Excluding video/computer games: FV: -0.11 (95%CI: -0.28 - -0.10; p=0.04) |
| **(60)** | Gender, ethnicity, father’s occupational status | *Selecting F and V ‘very often’ at home:*  Ethnicity*:* Black students more frequently ate V than Anglo and Mexican-American student (p<0.001). Mexican-American less frequently ate F than Black and Anglo student (p<0.001); Father’s occupational status*:* High status students more frequently ate F than middle and low status students (p<0.01)  *Selecting F and V ‘very often’ at school:*  Gender*:* Boys more frequently ate F (p<0.01) and V (p<0.01) than girls; Ethnicity: Black students more frequently ate V than Anglo/Mexican-American student (p<0.001); Father’s occupational status*:* Low status students more frequently ate F than middle and high status students (p<0.001) |
| **(61)** | Stages-of-change assessment (pre-contemplation, contemplation / preparation, action / maintenance)  Confounders: gender, age, race/ethnicity | Students in the action/maintenance stages consumed FV more frequently than students in the pre-action stages (p<0.001) |
| **(62)** | Type of snack meal (morning, afternoon, evening) | F was eaten most often as a morning snack (20.5%) compared to afternoon snack (10.0%) and evening snack (13.3%) |
| **(63)** | Grade  *Students’ reports*: F, J and V preferences, meal source  *Parents’ reports*: Gender, ethnicity, parental education | *Preferences:* F preferences were positively correlated with F consumption for both grades, genders and all ethnic groups (p<0.01 or 0.05, r=0.22-0.36). V preferences were positively correlated with V consumption for only 4^th^ graders, both genders and European and Mexican-Americans (p<0.001 or 0.05, r=0.14-0.31). *Grade:* 4^th^ grade students consumed more total F, J and V than 5^th^ grade students (p<0.001). *Gender:* Girls consumed more V than boys (p<0.05). *Parental education*: Students of parents with a high school education consumed more total FJV than students of parents with a college degree/higher education (0.64 servings, level of significance not reported). *Significant interaction effects:* V intake: Ethnicity by gender interaction – level of significance not reported. F intake: grade by family education and grade by family education by ethnicity - level of significance not reported. *Meal source:* 5^th^ grade students who only had snack-bar meals consumed less total FJV than 5^th^ grade students who had only access to school-lunch meals (p<0.001) |
| **(64)** | *Children’s reports:* Home availability of FJV, home accessibility of FJV  *Parents’ reports:* Home availability of FJV, home accessibility of FJV  Gender (stratified), preferences (stratified) | *Structural equation model coefficients:*  *Child-reported model for FJV consumption:*  Model 1a / full sample: availability: 0.21, p<0.05; accessibility: 0.16, p<0.05. Explained variance =10%  Model 1b / stratified by gender: Boys: availability NS; accessibility NS. Explained variance = 1%. Girls: availability: 0.41, p<0.001 / accessibility: 0.31, p<0.01. Explained variance = 35%  Model 1c / stratified by preferences: High preferences: availability: 0.19, p<0.05 / accessibility NS. Explained variance = 11%. Low preferences: availability: 0.28, p<0.05 / accessibility: 0.27, p<0.05. Explained variance = 23%  *Parent-reported model for FJV consumption:*  Model 2: parent-reported availability NS / parent-reported accessibility: 0.31, p<0.05. Explained variance = 8%  *Child- and parent-reported model for FJV consumption:*  Model 3: parent-reported availability NS / parent-reported accessibility: 0.21, p<0.05, child-reported availability: 0.22, p<0.05 / child-reported accessibility NS. Explained variance = 12% |
| **(65)** | *Students’ reports*: Food source  *Parents’ reports*: Gender, race/ethnicity | *Cohort 1*: Servings of F and regular V decreased 33% and 42% respectively (p<0.001) between year 1 to year 2. At year 1, the students only had access to national school lunch meal. At year 2 the students also had access to a snack bar  *Cohort 2*: Consumption of regular V decreased 10% (p<0.05) for children in middle school from 5^th^ to 6^th^ grade. F consumption did not change. Similar patterns were seen for both genders. Asian-American students had higher regular V intake (p<0.001) than African, European and Mexican-Americans. Cohort 2 attended middle school for both years, where snack bar/à la carte line was available. At 2 year follow-up cohort 2 students had lower F consumption compared with cohort 1 (p<0.05) |
| **(66)** | *Children’s reports:* age, number of meals, number of snacks, ate breakfast both days, sweetened beverage preferences, bottled water preference, social desirability  *Parents’ reports:* family income, material possessions, parental educational level, BMI, home availability of FJV, home accessibility of FJV, home high/low-fat food availability, mean home high/low-fat food practice, mean low-fat food barriers, mean FJV barriers | *Model A (BMI + demographic variables):* V: parental education was positively associated with V intake (p=0.043), lower BMI levels were associated with higher intake of V (p=0.002) (R^2^=0.136)  *Model B (psychosocial variables (availability/accessibility/barriers/preferences) + demographic variables):* J: parental education was positive associated with J intake (p=0.029); mean availability (p=0.020) and accessibility (p=0.019) of FJV were negatively associated with J intake; mean FJV barriers were positively associated with J intake (p=0.017) (R^2^=0.238) |
| **(67)** | Grade, gender, ethnicity, self-efficacy, preferences, outcome expectation | High F preferences were associated with high intake of F (p=0.01)  High V preferences (p=0.01) and high self-efficacy (p=0.04) were associated with high intake of V  High F preferences (p=0.03) and high V preferences (p=0.01) were associated with high intake of FV |
| **(68)** | Past week fast food restaurant use, gender (stratified)  Confounders: grade, race, SES (based on parental education, employment, student eligibility for free/reduced lunch and family receipt of public assistance), BMI, total energy intake | Among both boys and girls inverse associations were observed for fast food restaurant use and frequency of F consumption (p<0.001), V consumption (including French fries) (p<0.001), and V consumption (excluding French fries) (p<0.001) |
| **(69)** | Depressive symptoms, gender (stratified)  Confounders: race, grade | Among both boys and girls depressive symptoms were not significantly associated with daily servings of V or daily servings of F |
| **(70)** | Frequency of family dinner/supper  Confounders: age, gender, BMI, physical activity, hours of television watched, smoking intention, presence of smoking in the home, 2-parent home vs other arrangements, household income, frequency of the child making his or her own dinner, eat ready-made dinner | Odds ratio for eating 5 or more servings of FV a day by a one-category (most days vs. never or some days) increase in family dinner frequency = 1.45 (95%CI: 1.37-1.53, adjusted by age and gender)  No major changes were seen by further confounder control (effect sizes not reported) |
| **(71)** | Type of meal (breakfast, lunch, dinner, snacks) | Most FV were consumed at dinner, followed by lunch. No major difference in consumption was seen between breakfast and snacks |
| **(72)** | Parental intake of F and V, home FV availability, V being served at dinner, gender (stratified)  Confounders: school level (middle/high), parent SES (based on parental educational level, family income, and job status), parent gender, parent race/ethnicity | FV availability a home was positively associated with frequency of FV consumption among girls (*t*_trend_=2.70, p<0.01)  Parental intake of both F (*t*_trend_=3.17, p<0.01) and V (*t*_trend_=3.72, p<0.01) was positively associated with frequency of F and V intake among girls |
| **(73)** | Sample I: *Students’ reports:* day of consumption, F and V preferences, outcome expectations. *Parents’ reports:* Availability and accessibility of FJV at home  Sample II: Socio-economic status index (pct. of students at each school receiving free lunch). *Staff reports*: School food availability (number of food items served at school ) | *Study I:* Home availability/accessibility was moderately related to both F and V weekday and weekly consumption, and to total FV weekday, weekly, and weekend consumption (β-values: 0.05-0.08, p-values:0.02-0.07) also when adjusted for preferences and outcome expectations. FV preferences were positively associated with consumption (no data provided)  *Study II*: Food availability in school was moderately related to consumption of F/J (r=0.51, p<0.01), high fat V (r=0.32, p<0.05) and for all FV (r=0.28, p<0.01) when adjusting for pct. of students at school receiving free school lunch  Schools with higher pct. of students receiving free school lunches served fewer servings of F (r=-0.41, p<0.01) and their students ate fewer servings of F (r=-0.34, p<0.05) and more servings of V and legumes (r=0.53, p<0.001). Number of F/J items served at school accounted for 25 % of the variance in the servings of F and J consumed adjusted for socio-economic status |
| **(74)** | Gender, race, SES (median income of the census tract in which the student reside), body fat class | No significant associations |
| **(75)** | *Students’ reports:* knowledge, modelling (parent intake), self-efficacy, outcome expectations  *Parents’ reports:* home availability of FV | Home availability of FV was positively associated with frequency of FV intake (p=0.02)  *Path coefficients:*  *Medium/high availability*: parent intake =0.10, p<0.05; child knowledge =0.13, p<0.05; parent knowledge (indirect) =0.02, p<0.10; parent self-efficacy (indirect) =0.02, p<0.10  *Low home availability of FV*: child self-efficacy =0.12, p<0.10  Explained variance = 2% |
| **(76)** | *Individual level (students’ reports):* Confounders: gender, race/ethnicity, participation in the free/reduced lunch program, number of parents at home, highest level of education for mother and father, number of parents working full-time.  *School level (observations):* Availability of à la carte programmes, number of snack vending machines, number of beverage vending machines, fried potatoes served with school lunch program | School à la carte program availability was negatively associated with total daily intake of F (p=0.005) and total servings of FV (p=0.02). Snack vending machines were negatively related to the average total daily servings of F consumed. For each vending machine present in a school, students’ mean intake of F servings declined by 11% (p=0.03). Fried potatoes being served to students at school lunch was positively associated with average total daily V intake (including potatoes) (p=0.004) and total daily V and F intake (p=0.009) |
| **(77)** | Gender, age, cultural groups, having one or more school lunches | The Spanish-American children consumed less V than the Anglos  Having school lunch improved the intake of F and V |
| **(78)** | Race, gender (stratified), type of meal (stratified) | White boys more frequently ate F than Black boys (p≤ 0.05). White boys more frequently ate F for breakfast (p≤ 0.05) and snacks (p≤ 0.05), and V for lunch (p≤ 0.05) than Black boys. White girls more frequent ate F (p≤0.05) and V (p≤ 0.05) for snacks |
| **(79)** | Intentions, attitudes, subjective norms, barriers, gender (stratified), SES (based on receiving free/reduced lunch, parent’s educational level, work status)(stratified) | Standardised path coefficient = β: The model with the best fit showed both direct (β=0.20) and indirect (β=0.33) effects of barriers on frequency of FV intake, but only indirect effects from attitudes (β=0.13) and subjective norms (β=0.34) mediated through intentions (β=0.11). Explained variance = 7% of FV intake  Gender showed moderating effect: Model for boys: attitude → intention β=0.07; intention → FV intake β=0.16. Explained variance = 9% of FV intake. Model for girls: attitude → intention β=0.18; intention → FV intake β=0.06. Explained variance = 6% of FV intake. SES showed no moderating effect |
| **(80)** | Educational level of most educated parent, family income, age, gender, school status, race/ethnicity | *Eating less than 5 FV a day:*  Age: older adolescents were more likely to consume a diet low in FV than younger adolescents, β = 0.126, p<0.001 Educational level of parents: adolescents were less likely to consume a diet low in FV by increasing years of education:  β = -0.053, p=0.006  Race/ethnicity: intake did not differ among Black, White, and Hispanic adolescents. Non-black and non-Hispanic minority adolescents were less likely than white adolescents to consume a diet low in FV: β = -0.847, p<0.001 |
| **(81)** | TV viewing (hours per day)  Confounders only: grade, gender (stratified), race/ethnicity (stratified) | TV viewing was associated with eating insufficient FV among the total student population (OR=1.36, p<0.05) and among White female (OR=1.90, p<0.05) and White male (OR=1.59, p<0.05) students. Among Hispanic males, TV viewing was related inversely to insufficient consumption of FV (OR=0.56, p<0.05) |
| **(82)** | *Socio-demographic variables*: gender, date of birth, race/ethnicity, free/reduced-cost meal program, number of parents with whom they live, highest level of educational attainment for each parent, number of parents working full time  *Socio/environmental domain*: subjective norms on healthful eating, perceived barriers, parenting style, outlook for future  *Individual domain*: valuation of health/appearance/achievement, outcome expectations, nutrition knowledge, spiritual beliefs in health behaviours, depression  *Behaviour domain:* preferences | *Frequency of FV consumption (servings/day) (ERR=event rate ratio):*  High subjective norms: ERR range from 0.94 (10^th^) to 1.07 (90^th^)  High maternal authoritative parenting style: ERR range from 1.06 (75^th^) to 1.17 (90^th^)  High paternal non-authoritative parenting style: ERR range from 0.94 (10^th^) to 1.09 (90^th^)  Low nutritional knowledge: ERR range at the 25^th^ percentile = 1.24 compared to the median)  Strong influence of spirituality: ERR range from 0.94 (10^th^) to 1.05 (90^th^)  Being depressed: ERR range from 0.95 (10^th^) to 1.12 (90^th^)  Healthy preferences: ERR range from 0.82 (10^th^) to 1.22 (90^th^)  Parent educational level: both parents have finished high school or less – ERR = 0.81 (reference = both parents have finished college or more)  All associations: p<0.05  Approximated explained variance = 31% |
| **(83)** | TV viewing while eating, day (weekday/weekend day) | *Sample 1 (grade 3):*  *Pct. of energy from F and V:* TV on/off: lowest consumption of V when TV on (p<0.0001). TV viewing (on/off) x day (i.e. weekday or weekend day): F was less likely to be consumed during television viewing on weekdays than on weekend days (p<0.05)  *Sample 2 (grade 5):*  *Pct. of energy from F and V:* TV on/off: lowest consumption of V when TV on (p<0.0001). Day (weekday/weekend days): higher consumption of V (p<0.05) on weekend days and lower consumption of F (p<0.01) on weekend days |
| **(84)** | Gender, household SES (no parent working, eligibility for free/reduced-price school lunch, use of federal assistance programs), race/ethnicity, family structure, meals prepared by the subject/other child, skipped meals, school lunch participation | *Lowest score on 5 A Day index:*  2^nd^ graders: skipping meals, not participating in school lunch programs  5^th^ graders: being from medium/high SES households, not being Black non-Hispanic (they had the highest score), being from single-parent households, consuming meals prepared by a child, skipping meals, not participating in a school lunch programme. All associations: p<0.02  Some differences in consumption by SES and race/ethnicity were mediated by participation in school lunch |
| **(85)** | *Socio-demographic variables:* Parental education/employment, age, gender, race/ethnicity  *Behaviours:* Dieting frequency, binge eating, tobacco use, alcohol use, marijuana use, past suicide attempts  *Psychosocial variables*: Weight satisfaction, academic achievement, family connectedness  BMI | *Socio-demographic variables and BMI:*  *Inadequate F / V:*  Low SES: OR=2.03/1.59, p<0.0001; African-American: OR=0.73/1.73, p<0.0001; Female: OR=0.91/0.91, p<0.001; BMI>23.8: OR=1.20/NS, p<0.0001  *Psychosocial and behavioural variables controlled for socio-demographic variables and BMI:*  *Inadequate F / V:*  Weight dissatisfaction: OR=1.35/1.30, p<0.0001; Poor school achievement: OR=1.59/1.68, p<0.0001; Low family connectedness: OR=2.06/2.05, p<0.0001; Dieting: OR=NS/1.27, p<0.001; Binge eating: OR=1.17/1.20, p<0.001; Tobacco use: OR=1.54/1.59, p<0.001; Alcohol use: OR=1.22/1.39, p<0.001; Marijuana use: OR=1.33/1.56, p<0.001; Suicide attempts: OR=1.30/1.40, p<0.001 |
| **(86)** | *Socio-environmental variables*: social support for healthy eating, family meal patterns, food security, home availability of F and V, family socioeconomic status (primarily parental level of education)  *Personal variables*: preferences, health/nutrition attitudes, weight/body concerns, BMI, self-efficacy  *Behavioural variables*: meal frequency, weight control behaviours, fast food intake | *Path coefficients for servings of FV per day:*  Direct effects: preferences =0.13, home availability =0.29  Indirect effects through preferences: health/nutrition attitudes =0.45  Indirect effects through home availability: social support for healthy eating =0.38, family meal patterns = 0.10, food security =0.31, family socioeconomic status =0.13  All p-values < 0.01  Explained variance = 13%  Interaction: preferences x home availability (p<0.001) |
| **(87)** | Family meals  Confounders: gender, school level, race, SES (parental educational level) | Frequency of family meals (past week) was positively associated with frequency of F, V, and FV intake (p(trend)<0.001) |
| **(88)** | Type of breakfast (home/school) | F were more frequently consumed at school breakfast than home breakfast |
| **(89)** | Type of school (public/private), gender (stratified) | Students from private schools were more likely to have eaten F on the previous day than students from public schools (females: p<0.001, males: p<0.05). Females from private schools were more likely to have eaten FJ than females from public schools (p<0.001). Students from private schools were more likely to have eaten green salad than students from public schools (females: p<0.001, males p<0.001). Students from private schools were more likely to have eaten cooked V than students from public schools (females: p<0.001, males: p<0.01) |
| **(90)** | Gender, race, grade (stratified) | *2-4 servings of F per day*: In both grade 8 and 11 White students ate more F than African-American students (p<0.05)  *3-5 servings of V per day*: In grade 8 African-American students ate more V than White students (p<0.05) |
| **(91)** | Gender, parental education, age, self-efficacy, outcome expectations, preferences, exposure, social norms (regarding perception of family and peer attitudes towards F and V intake), health knowledge, asking skills/behaviour | *Number of servings of FV/week:*  Preferences (positive association, p<0.01); outcome expectations (positive association, p<0.01). Explained variance = 10%  *Number of servings of FV/meal:*  Preferences (positive association, p<0.01); Weekend x preferences (p<0.01); Meal x preferences (p<0.01)  Explained variance = 11% |
| **(92)** | Gender, ethnicity, age (confounder) | *FV*: Gender (Georgia: girls ate more than boys (p<0.01)); ethnicity (Georgia: African-Americans ate more than European-Americans (p<0.10)). *F:* Gender (Georgia: girls ate more than boys (p<0.05)); ethnicity (Georgia: African-Americans ate more than European-Americans (p<0.05); Minnesota: Asian-Americans/Pacific Islanders, African-Americans ate more than European-Americans (p<0.05)). *V:* Gender (Georgia: girls ate more than boys (p<0.05)); ethnicity (Georgia, European-Americans ate more than Asian-Americans/Pacific Islanders who ate more than African-Americans (p<0.05)) |
| **(93)** | *Students’ reports*: gender, motivation (self-efficacy, outcome expectations, food preferences), knowledge, nutritional education, modelling *Parents’ reports*: home availability of F and V | *Path coefficients for servings of FV/day (p<0.05):*  *Split 1 (to develop model):* Direct effects: availability =0.08; motivation =0.10. Explained variance = 11%  *Split 2 (to confirm model):* Direct effects: motivation =0.09; knowledge =0.26. Indirect effects: availability through motivation. Explained variance = 11%  The best fit was achieved for split 1  *Males:* Direct effects: motivation =0.19. Indirect effects: availability through motivation; availability through motivation and knowledge. Explained variance = 17%  *Females:* Direct effects: availability =0.09; motivation =0.08; knowledge =0.24. Explained variance= 12%  All p-values < 0.01 |
| **(94)** | Meal pattern (consistent / inconsistent) | The number of FV consumed was highest among adolescents with consistent meal patterns (p<0.01) |
| **(95)** | Working status of adolescent | Non-working adolescents eat more V as part of the evening meal than working adolescents (p=0.07) |
| **(96)** | Consumption of fried food away from home  Confounders: age, gender | Frequency of F and V consumption was negatively associated with frequency of consuming fried food away from home (p<0.0001) |
| **(97)** | TV/video watching, computer use, reading/homework, gender (stratified)  Confounders: race, SES (parental education level), age | For both boys and girls high television/video use was associated with less frequent consumption of both F and V (p<0.05). Reading/homework was associated with more frequent consumption of both F and V among both boys and girls (p<0.05). Computer use was also associated with more frequent consumption of both F and V among girls (p<0.05), but not among boys |
| **(98)** | Gender, race/ethnicity, age, parental education, body weight perception, parent present when student leave for school, parent present when student return from school, food decision-making, family meals | *Not eating 2+ V/day:*  Race/ethnicity: reference=White, Black: OR=1.29 (p<0.01), Hispanic: OR=1.49 (p<0.001), Asian: OR=0.69 (p<0.01); Parental education: reference=high school degree, some education after high school: OR=0.80 (p<0.001), college degree: OR=0.73 (p<0.001); Body weigh perception: reference=‘about the right weight’, overweight: OR=1.10 (p<0.05); Family meals: reference=three or fewer family meals/week, six or seven family meals/week: OR=0.62 (p<0.001), four or five family meals/week: OR=0.81 (p<0.01)  *Not eating 2+F /day:*  Age: OR = 1.03 (p<0.05); Race/ethnicity: reference=White, Black: OR=0.67 (p<0.001), Hispanic: OR=0.73 (p<0.001), Asian: OR=0.50 (p<0.001); Parental education: reference=high school degree, some education after high school: OR=0.83 (p<0.05), college degree: OR=0.62 (p<0.001); Body weigh perception: reference=‘about the right weight’, overweight: OR=1.14 (p<0.001); Family meals: reference=three or fewer family meals/week, six or seven family meals/week: OR=0.69 (p<0.001), four or five family meals/week: OR=0.78 (p<0.001) |
| **(99)** | SES (participation in free/reduced-price school lunch, both parents not working, or received social or food assistance), lunch source, grade, gender, race, family structure, mother employed/not employed | Lunch source: students bringing lunch from home ate less FV than those eating a school lunch (p<0.001)  Family structure: children in single-parent households were less likely to have eaten V than those living in two-parent households (β = -0.33, p<0.05) |
| **(100)** | Gender, race/ethnicity, parent’s education, family income  Confounder: age | Girls more likely met the recommendation for both F and V than boys, p<0.001. Fewer Hispanic-Whites than Non-Hispanic-Whites, Blacks, and Asians met the recommendations for V, p<0.01. Fewer Non-Hispanic-Whites than Hispanic-Whites, Blacks, and Asians met the recommendation for F, p<0.05. Students whose parents received higher education had highest consumption of F and V, p<0.05 |
| **(101)** | Gender, race, grade, perceived weight, family communication, family monitoring, family structure, hours spent at home without parents | *High frequency of FV intake:*  High levels of family communication (p<0.05, R^2^ = 0.021); High levels of parental monitoring (p<0.05, R^2^ = 0.049); As family structure moved from two parent to foster care the pct. of adolescents eating one or more FV per day decreased (p<0.05); A decrease in the pct. of adolescents eating one or more FV per day was observed if the adolescent spent 5 or more hours at home without an adult (p<0.05). Asian adolescents more frequently ate FV than White, Hispanic, and Black adolescents (level of significance not reported) |
| **(102)** | Authoritative parenting, parent control, parent modelling, parent support, F/V home availability, self-efficacy  Confounders: grade, gender, race, SES (free/reduced school meals), rural/urban schools | Model 1 / including the four parent variables: parent modelling and parent support were positively associated with frequency of FV intake (p<0.001, R^2^ = 0.227)  Model 2 / including the four parent variables + self-efficacy and FV availability: Parent modelling, self-efficacy and F/V availability were positively associated with frequency of FV intake (p<0.001, R^2^ = 0.392)  The effect of parent support was completely mediated by self-efficacy  Moderating effect of FV availability: parent support had a positive effect on FV consumption for students with low FV availability (p=0.019); parent modelling had a positive effect on FV consumption for students with high FV availability (p=0.02) |
| **(103)** |  |  |
| **(104)** | Household income, gender (stratified)  Confounder: age | *Not consuming F:*  Boys/Household income: level 5 (high) OR=1.0; level 4 OR=1.6; level 3 OR=1.4; level 2 OR=3.5; level 1 (low) OR=3.0; p-value for significance of model < 0.01  *Mean variety of F:* NS |
| **(105)** | SES (urban index of relative advantage ranking), gender | Gender: Males ate both F and V less frequently than females (p<0.05)  SES / stratified by gender: Females from high SES groups had higher frequency of both F and V consumption than low SES females (p<0.05) |
| **(106)** | Gender | F (p=0.0005) and green or yellow V (p=0.003) were consumed more often by girls than boys |
| **(107)** | Gender, meal type (stratified) | *Lunch:* F was eaten more by girls (40%) than by boys (27%) |
| **(108)** | Gender, family income | *Analyses of frequency of consumption of approximately 28 different items of F and V:*  For three F-items and seven V-items more consumers were found among girls than boys (p<0.10-0.0001). For pure FJ and five types of V more consumers were found among adolescents from high family income groups than among adolescents from low family income groups (p<0.10-0.0001) |
| **(109)** | Gender, liking, perceived healthiness, perceived parental intake, perceived friends’ intake, grade (stratified) | Girls ate more apples than boys (p<0.01)  Liking was associated with eating apples, and tomatoes at least once a week (p<0.01). For tomatoes this was observed for both boys and girls, and among 7/8 graders and 9/10 graders  Perceived parents’ usage was associated with drinking orange J (p<0.01) for both boys and girls and among 7/8 graders and 9/10 graders |
| **(110)** | Gender, age, height, weight, fatness, exercise-level, alcohol use, cigarette smoking, analgesics and vitamin-supplements, school type, educational level of father/mother, social status (not specified), family size | Gender: boys ate more FV than girls (p<0.01)  Age: among boys, increasing age was associated with higher intake of FV (p<0.01) |
| **(111)** | Gender, age, height, weight, fatness, exercise-level, alcohol use, cigarette smoking, analgesics and vitamin-supplements, school type, educational level of father/mother, social status (based on occupation of main income-earner), family size | Age: among boys, increasing age was associated with higher intake of FV (p<0.01)  Students 12 years old or younger: exercise was positively associated with FV intake (p<0.01); increasing fatness was associated with decreasing intake of FV (p<0.01) |
| **(112)** | Gender, age, height, weight, fatness (BMI), alcohol use, cigarette smoking, vitamin supplements and analgesics, exercise, job status of main income earner, father’s education, mother’s education, family size, school type | *Whole sample*: intake of citrus and berries increased with increasing weight (p<0.01); intake of green V increased with increasing intake of vitamins (p<0.001); intake of citrus and berries was lowest at state and rural schools (p<0.01) and highest at catholic schools (p<0.01); intake of potatoes was highest at rural schools (p<0.01); intake of nuts and legumes was highest at remote schools (p<0.01); intake of potatoes decreased by increasing educational level of mother (p<0.01); intake of citrus and berries increased by increasing job status of main income earner (p<0.01)  *Boys:* intake of potatoes (p<0.001) and nuts and legumes (p<0.01) increased by increasing age; intake of citrus and berries increased by increasing weight (p<0.001); intake of citrus and berries was lowest at state schools (p<0.01); intake of citrus and berries increased by increasing job status of main income earner (p<0.01)  *Girls:* intake of potatoes decreased by increasing fatness (p<0.01); intake of potatoes was highest at rural schools (p<0.001); intake of citrus and berries increased by increasing job status of main income earner (p<0.001); intake of green V increased by increasing educational level of mother (p<0.01) |
| **(113)** | Preferences, perceived healthfulness, descriptive social norms (perceived parental consumption and perceived friend consumption) | *Number of days of usage in an average week:*  *Apple*: liking: β=0.44, p <0.0001; parents’ consumption: β=0.12, p<0.0001  *Orange J*: liking: β=0.22, p<0.0001; parents’ consumption: β=0.37, p<0.0001; friends’ consumption: β=0.06, p<0.01  *Potato*: liking: β=0.22, p<0.0001; parents’ consumption: β=0.33, p<0.0001; friends’ consumption: β=0.08, p<0.001  *Tomato*: liking: β=0.51, p<0.0001; parents’ consumption β=0.13, p<0.0001 |
| **(114)** | Gender, urbanisation | *Frequency of F and V consumption:*  Urban adolescents consumed fewer F (p=0.003) and V (p=0.000) than rural adolescents. Females consumed fewer F (p=0.000) and V (p=0.000) than males |
|  |  |  |
|  |  |  |

*based on children’s / students’ reports if nothing else specified

F = fruit; V = vegetables; J = juice, FJ = fruit juice; FJV = fruit, juice and vegetables; OR = Odds ratio; NS = non-significant; pct. = percentage; CI = Confidence interval

|  |  |  |
| --- | --- | --- |
|  |  |  |
|  |  |  |
|  |  |  |
|  |  |  |
|  |  |  |
|  |  |  |
|  |  |  |
|  |  |  |
|  |  |  |
|  |  |  |
|  |  |  |

|  |  |  |
| --- | --- | --- |
|  |  |  |
|  |  |  |
|  |  |  |
|  |  |  |
|  |  |  |

|  |  |  |
| --- | --- | --- |
|  |  |  |
|  |  |  |
|  |  |  |
|  |  |  |
|  |  |  |
|  |  |  |
|  |  |  |
|  |  |  |
|  |  |  |

|  |  |  |
| --- | --- | --- |
|  |  |  |
|  |  |  |
|  |  |  |
|  |  |  |
|  |  |  |
|  |  |  |

|  |  |  |
| --- | --- | --- |
|  |  |  |
|  |  |  |
|  |  |  |
|  |  |  |
|  |  |  |
|  |  |  |
|  |  |  |
|  |  |  |
|  |  |  |
|  |  |  |
|  |  |  |
|  |  |  |
|  |  |  |
|  |  |  |
|  |  |  |
|  |  |  |
|  |  |  |
|  |  |  |
|  |  |  |
|  |  |  |
|  |  |  |
|  |  |  |
|  |  |  |

|  |  |  |
| --- | --- | --- |
|  |  |  |
|  |  |  |
|  |  |  |
|  |  |  |
|  |  |  |
|  |  |  |

|  |  |  |
| --- | --- | --- |
|  |  |  |
|  |  |  |
|  |  |  |
|  |  |  |
|  |  |  |

|  |  |  |
| --- | --- | --- |
|  |  |  |
|  |  |  |
|  |  |  |
|  |  |  |
|  |  |  |
|  |  |  |

|  |  |  |
| --- | --- | --- |
|  |  |  |
|  |  |  |
|  |  |  |
|  |  |  |
|  |  |  |

|  |  |  |
| --- | --- | --- |
|  |  |  |
|  |  |  |
|  |  |  |
|  |  |  |
|  |  |  |
